# The gaps and challenges in digital health technology use as perceived by patients: a scoping review and narrative meta-synthesis

**DOI:** 10.3389/fdgth.2025.1474956

**Published:** 2025-03-27

**Authors:** Georgia Livieri, Eleni Mangina, Evangelos D. Protopapadakis, Andrie G. Panayiotou

**Affiliations:** ^1^ Department of Rehabilitation Sciences, School of Health Sciences, Cyprus University of Technology, Limassol, Cyprus; ^2^ Computer Science and Informatics, School of Computer Science, University College Dublin, Dublin, Ireland; ^3^ Department of Philosophy, School of Philosophy, National and Kapodistrian University of Athens, Athens, Greece

**Keywords:** digital health, patients, digital healthcare technology, gaps, challenges, ethical framework, human rights

## Abstract

**Introduction:**

Digital health has revolutionized the landscape of healthcare through personalized care, moving away from the traditional approach of treating symptoms and conditions. Digital devices provide diagnostic accuracy and treatment effectiveness while equipping patients with control over their health and well-being. Although the growth of technology provides unprecedented opportunities, there are also certain issues arising from the use of such technology. This scoping review aimed to explore perceived gaps and challenges in the use of digital technology by patients and meta-synthesize them. Identifying such gaps and challenges will encourage new insights and understanding, leading to evidence-informed policies and practices.

**Methods:**

Three electronic databases were searched (Cinahl EBSCO, Pubmed, and Web of Science) for papers published in English between January 2010 and December 2023. A narrative meta-synthesis was performed. The review followed the Preferred Reporting Items for Systematic Reviews and Meta-Analysis (PRISMA) 2009 checklist.

**Results:**

A total of 345 papers were retrieved and screened, with a noticeable increase in publication numbers after 2015. After the final selection, a total of 28 papers were included in the final meta-synthesis; these were published between 2015 and 2023. A total of 99 individual reports were included in the synthesis of these papers, comprising 25 identified gaps and 74 challenges.

**Discussion:**

Our meta-synthesis revealed several gaps and challenges related to patients' use of digital technology in health, including generational differences in digital propensity and deficiencies in the work process. In terms of ethics, the lack of trust in technology and data ownership was highlighted, with the meta-synthesis identifying issues in the realm of disruption of human rights. We, therefore, propose building a model for ethically aligned technology development and acceptance that considers human rights a crucial parameter in the digital healthcare ecosystem.

## 1 Introduction

Digital health has transformed healthcare by enabling real-time personalized monitoring and therapeutic care, moving away from the traditional “one-size-fits-all” approach (1). There is no doubt that digital health has shifted the paradigm of quality medical care (2). The ubiquitous availability of smartphones, wearable devices, and tablet computers and widespread internet connectivity have led to significant changes in human-technology interactions (3). Worldwide, each step of the patient care pathway is being improved by digital devices (1), as they have the potential to enhance our ability to diagnose diseases accurately, provide effective treatment, and improve healthcare delivery for individual patients while empowering patients to have greater control over their health and make informed decisions regarding their well-being (4).

The COVID-19 pandemic accelerated the adoption of digital health technologies, demonstrating the ability of such technologies to meet the needs of the population (5). Specifically, the pandemic has emphasized the crucial role of digital technologies in the healthcare sector (6), with many people being dependent on the internet and digital devices to access medical services and treatments (7). Healthcare systems are gradually realizing that modern technologies can optimize the patient journey, from symptom identification to long-term care, widening access to healthcare provision, reducing costs, and providing services tailored to individual needs (1). Nevertheless, there seems to be a mismatch between expectations for digital technology use in healthcare from both patients and developers and patients' own reported experiences (8). This mismatch is likely to result in the underuse of such technology.

Therefore, the main objective of this study was to identify and map in a systematic way the perceived challenges and gaps in the use of digital health services by patients, as reported in the published literature. The identified gaps and challenges have been compiled, categorized, and meta-synthesized to encourage new insights and understanding, leading to evidence-informed policies and practices. This meta-synthesis offers a unique contribution to digital health technology by integrating diverse findings to provide a comprehensive perspective on its broader implications for healthcare systems, policy, and patient outcomes. It develops a structured ethical framework grounded in human rights principles, guiding stakeholders in addressing ethical challenges such as patient rights, equity, and data privacy. Additionally, it identifies underexplored risks, such as the dehumanization of patient-provider interactions, and proposes human-centered solutions. The review also highlights the need for region-specific strategies, particularly in low- and middle-income countries. By offering holistic insights and practical recommendations, this synthesis provides guidance for policymakers, healthcare providers, and technology developers to foster ethical, inclusive, and patient-centered digital health solutions.

## 2 Methods

This study adopts a systematic mapping design as a robust methodological approach to establish the extent of evidence (2) on identifying the challenges and illuminating the gaps raised by digital technology use in patients. The guidelines and criteria set in the Preferred Reporting Items for Systematic Review and Meta-Analyses (PRISMA) protocol (9) were used.

### 2.1 Search strategy

We initially performed a rapid synthesis of evidence with data mining via a few selected keywords in the EBSCO CINAHL database, including the terms “digital health”, “gaps and challenges” and “patients”. This exercise served as a preliminary measure of exploring research activities in the fields of interest and guided a more targeted search by indicating arbitrary boundaries of reviews that were within the time and practical constraints of this study (10). Consequently, we used the Population, Intervention, Comparison, and Outcome (PICO) format for systematic reviews (9) in order to identify the search items/categories that guided the structured searches. The search terms were carefully chosen to balance specificity and comprehensiveness. The search categories included “digital health”, “telehealth”, “mobile health”, “electronic health”, “patients”, “gaps” and “challenges”, which were used in different combinations (see Table 1) with Boolean operators (AND, OR) to capture studies addressing both the technology and its impacts on healthcare delivery in order to maximize relevant literature retrieval. These terms were further refined to focus on studies published from 2010 onwards (based on the initial exploration) to ensure the review reflects recent trends and developments in the field. This approach ensured a systematic exploration of digital health and patient experiences, providing a structured foundation for our search, enhancing transparency and strengthening the validity of our findings.

**Table 1:** Structured search strategy and key terms.

Research question
What are the gaps and challenges in health digital technology use as perceived by patients?

No.	Conceptual Framework	Synonyms
#1	Digital health	• digital health or electronic health or ehealth
#2	Telehealth	• telehealth or telemedicine or personalized medicine or precision medicine or telemonitoring or telepractice or telenursing or telecare or ehealth or e-health or mhealth or digital health or technology
#3	Mobile health	• mobile health applications or wearable devices or mhealth or mobile apps or mobile health technology
#4	Electronic health	• electronic health records or electronic medical records or emr or ehr or patient medical record
#5	Gaps and challenges	• development or validation or effectiveness or intervention or approach or gaps or challenges or impact or evolution or frameworks
#6	Patients	• patients or patient or clients or client or individuals or individual or service user or service users

Final search	Final search string
	((#1) AND (#2) AND (#3) AND (#4) AND (#5) AND (#6))

A comprehensive search algorithm (see Table 2) was used to search three databases, EBSCO CINAHL, Pubmed, and Web of Science, modified as per the guidelines for each database. This structured approach ensures that our literature search captures a broad yet focused selection of studies. By clearly defining the search strategy, Table 2 enhances reproducibility and methodological transparency, allowing future research to build upon our findings. The search strategy was designed to be comprehensive and systematic. Databases such as PubMed, Cinahl and Web of Science were selected due to their breadth and relevance to healthcare and digital health research. The rationale for choosing these databases was to ensure that the review captured a wide range of peer-reviewed articles across disciplines, including medicine, healthcare technology, and policy. The searches were run from April to June 2021 for papers published between January 2010 and April 2021, with an updated search occurring between January 2021 and December 2023 for papers published between January 2021 and December 2023. The search was limited to full-text papers published in English.

**Table 2:** Database-Specific search algorithms for identifying patient-related digital health studies.

Database	Algorithm/strategy
EBSCO Cinahl	AB ((digital health or electronic health or ehealth)) AND AB ((mobile health applications or wearable devices or mhealth or mobile apps or mobile health technology)) AND AB ((telehealth or telemedicine or personalized medicine or precision medicine or telemonitoring or telepractice or telenursing or telecare or ehealth or e-health or mhealth or digital health or technology)) AND AB ((electronic health records or electronic medical records or emr or ehr)) AND AB ((development or validation or effectiveness or intervention or approach or gaps or challenges or impact or evolution or frameworks)) AND AB ((patients or patient or clients or client or individuals or individual or service user or service users))
Pubmed	(((((digital health[Title/Abstract] OR electronic health[Title/Abstract] OR ehealth[Title/Abstract]) AND (mobile health applications[Title/Abstract] OR wearable devices[Title/Abstract] OR mhealth[Title/Abstract] OR mobile apps[Title/Abstract] OR mobile health technology[Title/Abstract])) AND (telehealth[Title/Abstract] OR telemedicine[Title/Abstract] OR personalized medicine[Title/Abstract] OR precision medicine[Title/Abstract] OR telemonitoring[Title/Abstract] OR telepractice[Title/Abstract] OR telenursing[Title/Abstract] OR telecare[Title/Abstract] OR ehealth[Title/Abstract] OR e-health[Title/Abstract] OR mhealth[Title/Abstract] OR digital health[Title/Abstract] OR technology[Title/Abstract])) AND (electronic health records[Title/Abstract] OR electronic medical records[Title/Abstract] OR emr[Title/Abstract] OR ehr[Title/Abstract])) AND (development[Title/Abstract] OR validation[Title/Abstract] OR effectiveness[Title/Abstract] OR intervention[Title/Abstract] OR approach[Title/Abstract] OR gaps[Title/Abstract] OR challenges[Title/Abstract] OR impact[Title/Abstract] OR evolution[Title/Abstract] OR frameworks[Title/Abstract])) AND (patients[Title/Abstract] OR patient[Title/Abstract] OR clients[Title/Abstract] OR client[Title/Abstract] OR individuals[Title/Abstract] OR individual[Title/Abstract] OR service user[Title/Abstract] OR service users[Title/Abstract])
Web of Science	(((((AB = (digital health or electronic health or ehealth))) AND AB = (mobile health applications or wearable devices or mhealth or mobile apps or mobile health technology)) AND AB = (telehealth or telemedicine or personalized medicine or precision medicine or telemonitoring or telepractice or telenursing or telecare or ehealth or e-health or mhealth or digital health or technology))) AND AB = (electronic health records or electronic medical records or emr or ehr))) AND AB = (development or validation or effectiveness or intervention or approach or gaps or challenges or impact or evolution or frameworks))) AND AB = (patients or patient or clients or client or individuals or individual or service user or service users)

### 2.2 Inclusion and exclusion criteria

Papers were included if they fulfilled the following criteria: (1) were published between January 1st, 2010 (as initial searches identified a lack of literature on the topic prior to 2012) and December 27th, 2023, on EBSCO Cinahl, PubMed, or the Web of Science; (2) were published in the English language as a full-text paper; and (3) were relevant to the subject. Relevance was assessed as any mention of a gap or challenge, as defined below, based on the pre-identified eligibility criteria and PICO format:

Gap: “lack of”, “need for” and challenges: “barriers”, “fears”, “mismatches”, “desire for”, “risk”, “downsides”, “concerns”, “potential pitfalls”, and “limitations”.

The exclusion criteria were as follows: (1) not relevant to the study question; (2) books, e-books, news, magazines, reports, electronic resources, trade publications, conference material, dissertations/theses, nonprint resources, videos, or audios; (3) unavailable full texts in the English language; (4) catalog and non-peer-reviewed articles; (5) abstracts only papers; and (6) duplicates of the same study.

During the review process, studies were excluded based on a set of predefined criteria, including irrelevant focus: Studies that did not directly address digital health technologies or their impact on healthcare outcomes were excluded. This approach ensured that only studies with relevant findings were synthesized. A decision was made to include review papers as well, given their number but also so as not to miss any reported gaps or challenges.

### 2.3 Selection process and data extraction

The electronic search results were downloaded into a reference manager library (Mendeley Ltd., Elsevier). After duplicates were removed, titles and abstracts were screened initially by two authors (GL and AP) independently. Full-text copies of potentially relevant papers were retrieved and further assessed against inclusion/exclusion criteria. At both stages, any conflicts were resolved by consensus, and forwarded for content analysis.

Two authors (GL and AP) extracted the data independently and compared the results. The following data were extracted from each study: title, authors, publication year, country of study, study design if relevant, study population, type of technology reported and outcome (perceived experience of technology used), and any additional information deemed potentially useful or relevant for the meta-synthesis. Relevant data were extracted into a prespecified data extraction template in Microsoft Excel.

### 2.4 Critical appraisal

A risk of bias assessment was not deemed appropriate in this context because the aim was to identify and synthesize all the reported perceived challenges and gaps in the use of digital health technology by patients, as well as because one-third of the included papers were reviews.

While the literature review provided an extensive overview of existing studies, a more critical examination of the included sources is warranted. Each study reviewed has its strengths and limitations, and discussing these more explicitly can provide a clearer understanding of the quality of evidence and its implications for the findings of this meta-synthesis. For instance, several studies included in this review have small sample sizes or focus on specific demographic groups, which may limit the generalizability of their results. Others may suffer from methodological limitations, such as lack of longitudinal data or reliance on self-reported outcomes, which could introduce biases. Additionally, many studies were conducted in high-income countries, which may not fully capture the challenges faced by low- and middle-income countries (LMICs), potentially skewing the broader applicability of the conclusions. Nevertheless, due to the thematic analysis of the study, it was felt that any gap or challenge reported should be recorded and included in the meta-synthesis. No group or thematic category included reports from only one study.

### 2.5 Quality assessment and risk of bias

To ensure methodological rigor and transparency, we conducted a systematic quality assessment of the included studies (see Supplementary Table 1). Given the diverse study designs in our review, we employed an adapted quality appraisal approach suited to both qualitative and quantitative studies.

#### 2.5.1 Quality Assessment Criteria

Each study was evaluated based on the following criteria:

1. **Study Design and Methodology:** Clarity and appropriateness of the study design in addressing research questions.
2. **Data Collection Methods:** Use of validated instruments or standardized procedures for data collection.
3. **Analysis and Interpretation:** Rigor in data analysis, transparency in reporting findings, and discussion of limitations.
4. **Relevance to Review Objectives:** Direct alignment with the scope of identifying gaps and challenges in digital health adoption by patients.

### 2.6 Data synthesis

A thematic analysis of the papers was performed in a systematic, iterative manner. The process began with an initial coding phase, where key concepts and patterns were identified from the extracted study data. These initial codes were then grouped into broader themes through a process of refinement and re-categorization, guided by both the study's research questions and a review of relevant literature.

Following this structured approach, relevant gaps and challenges were first categorized conceptually using predefined terms to differentiate between gaps (e.g., “lack of,” “need for”) and challenges (e.g., “barriers,” “fears,” “mismatches,” “desire for,” “risk,” “downsides,” “concerns,” “potential pitfalls,” “limitations”). These were then further grouped into seven conceptual categories: (1) ethical issues, (2) educational/training issues, (3) usability issues, (4) communication issues, (5) functionality/structural issues, (6) technical issues, and (7) access issues.

To enhance reliability, two coders independently conducted the analysis, resolving any inter-coder discrepancies through regular discussions and consensus meetings. This ensured that the final themes accurately reflected the data and remained grounded in evidence.

As a final step, the seven categories were synthesized into three overarching pillars—(A) Digital Literacy, (B) Functionality/Usability, and (C) Trust—which provided the foundation for the meta-synthesis and final recommendations. The synthesis was performed by GL in discussion with all co-authors, ensuring a rigorous and transparent approach.

Our approach aligns with established meta-aggregation methodologies, particularly those outlined in recent systematic reviews and meta-synthesis studies [Zheng et al. (11); Zheng et al. (12)], as well as the JBI meta-aggregation framework [Lockwood et al. (13)]. In line with these methodologies, our study followed a structured process of data extraction, categorization, and synthesis to ensure a rigorous and transparent analysis. Specifically, (a) findings were extracted verbatim from each study and grouped into categories based on similarity in meaning; (b) these categories were further synthesized to produce higher-order synthesized findings, ensuring a more comprehensive interpretation of patient-reported gaps and challenges; and (c) the final meta-aggregation process led to the formulation of three overarching synthesized findings (pillars): Digital Literacy, Functionality/Usability, and Trust. By applying this structured synthesis approach, our study maintains methodological robustness and ensures that the final themes are deeply grounded in patient experiences and systematically derived from the literature.

## 3 Results

### 3.1 Search and selection results

**Figure 1:**
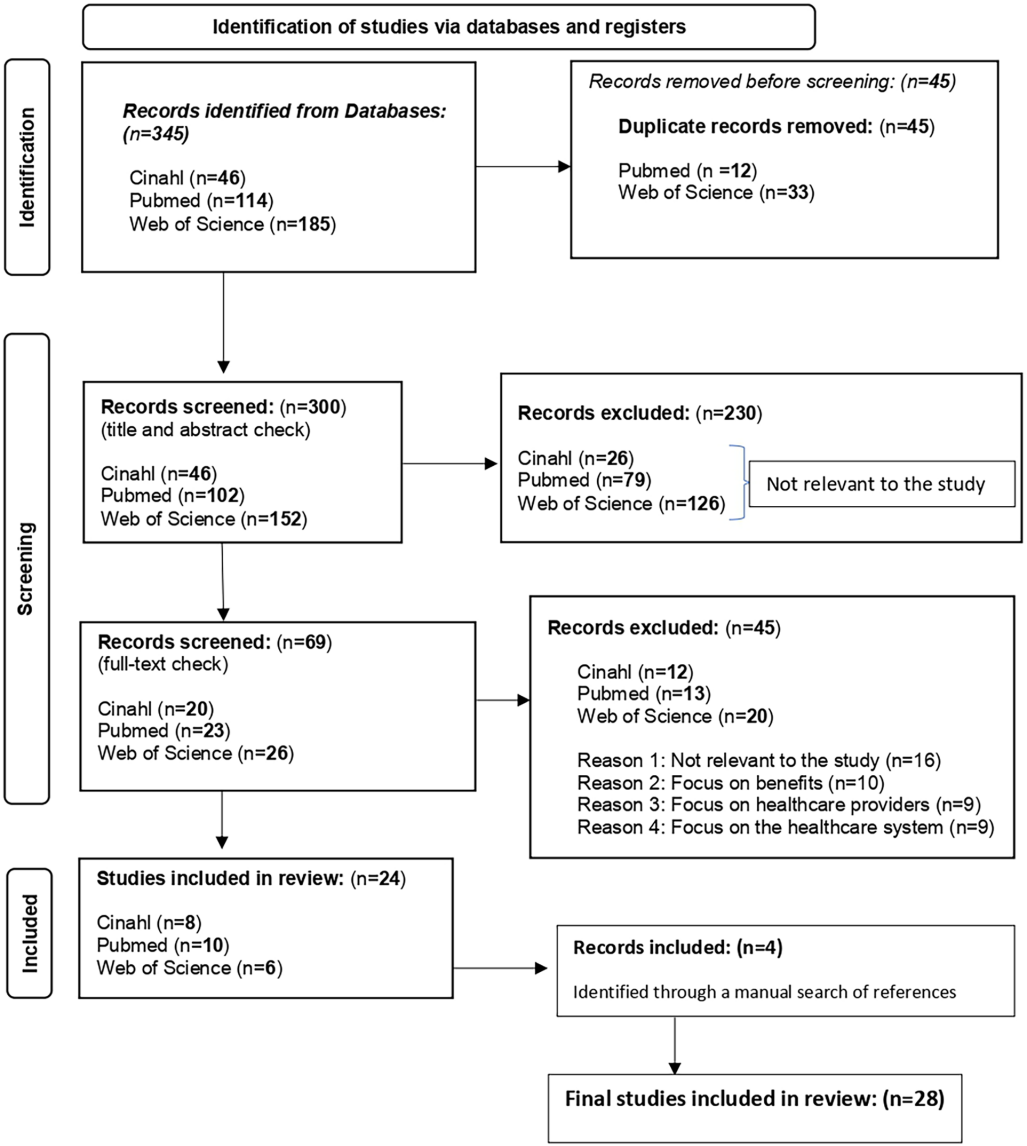
PRISMA flowchart of search outcome and patient study selection process.

The initial search resulted in a total of 345 papers. After a preliminary check, 45 duplicates were removed. The remaining 300 publications were screened by title and abstract (GL, AP), resulting in 69 full-text papers being included for full-text review. After a full-text review (GL, AP), a total of 24 (out of 69) papers were included in the final data extraction. Four additional studies were identified through a manual search of the included papers' references and included in the final data extraction, bringing the total number of studies included in the final review to twenty-eight (14). To ensure a transparent and systematic selection of studies, we employed the PRISMA framework to document the search outcome and screening process. Figure 1 illustrates the number of records identified, screened, excluded, and included in the final synthesis. This structured approach enhances the reproducibility of our methodology and ensures a rigorous evaluation of relevant literature.

### 3.2 Data mapping of the included papers

Data from all 28 included studies were extracted using a standardized tool (predefined Excel template) and mapped based on the thematic analysis described before. The rationale behind the taxonomy used was to highlight and correlate information first with regard to the characteristics of the included papers, including the year of publication, the country, the type of study, and the different types of digital technology; second, with regard to the categorization of the reported gaps and challenges.

### 3.3 Characteristics of the included papers

**Figure 2:**
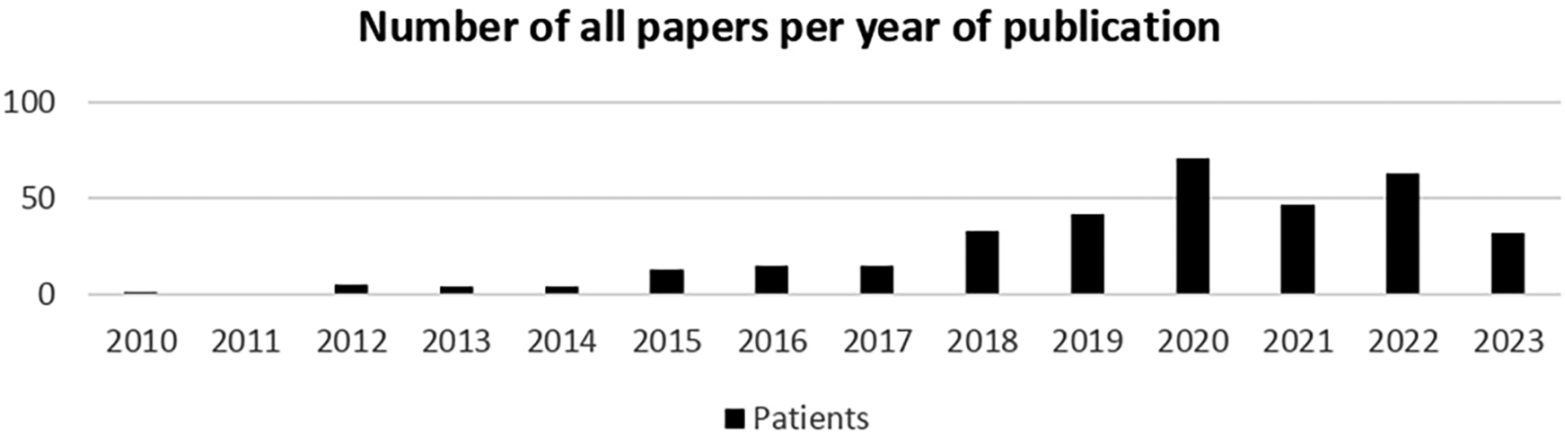
Annual trend of all identified papers on digital health and patient challenges (2010-2023).

**Figure 3:**
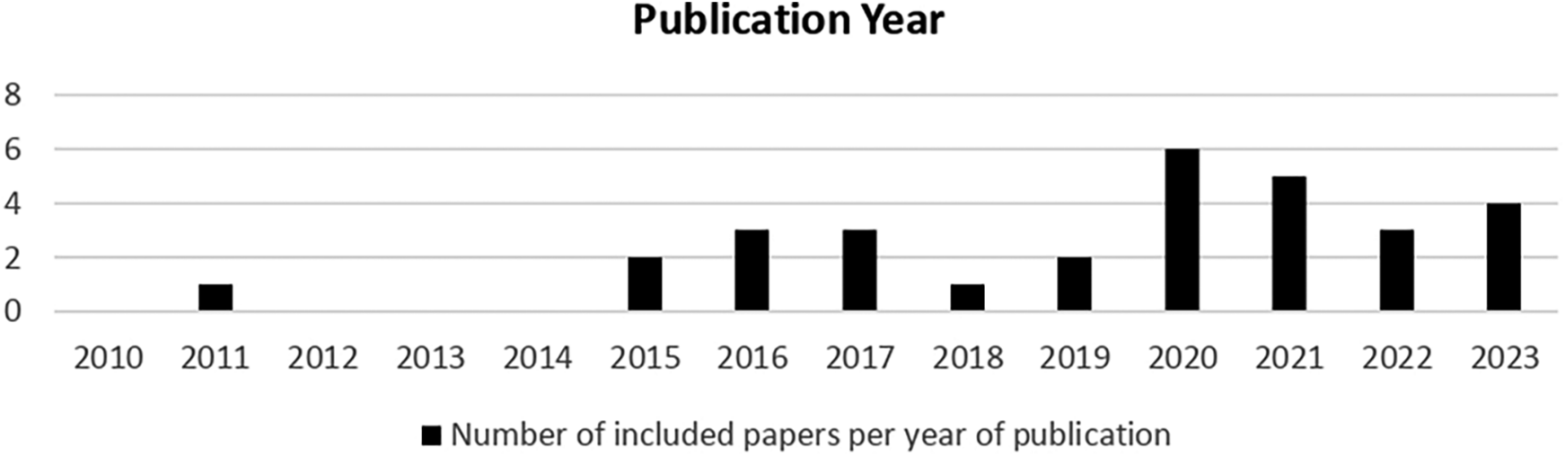
Annual distribution of included papers on digital health and patient challenges (2010-2023).

All included papers (*n* = 28) were published after 2015. When all 345 initially identified papers were included, there was an increasing trend in related topics since 2015, with a peak in 2020, perhaps indicating an increase in digital technology use by patients during the COVID-19 pandemic (Figures 2, 3). To assess the evolution of research on digital health and patient-related challenges, we analyzed the number of all identified papers published annually (Figure 2). This trend highlights the growing academic focus on digital health over time, reflecting increasing recognition of its impact on patient experiences and healthcare systems. As shown in Figure 3, the number of included studies increased steadily, peaking in 2020. This trend aligns with the broader surge in digital health research (Figure 2) and likely corresponds to the heightened adoption of digital technologies by patients during the COVID-19 pandemic.

**Figure 4:**
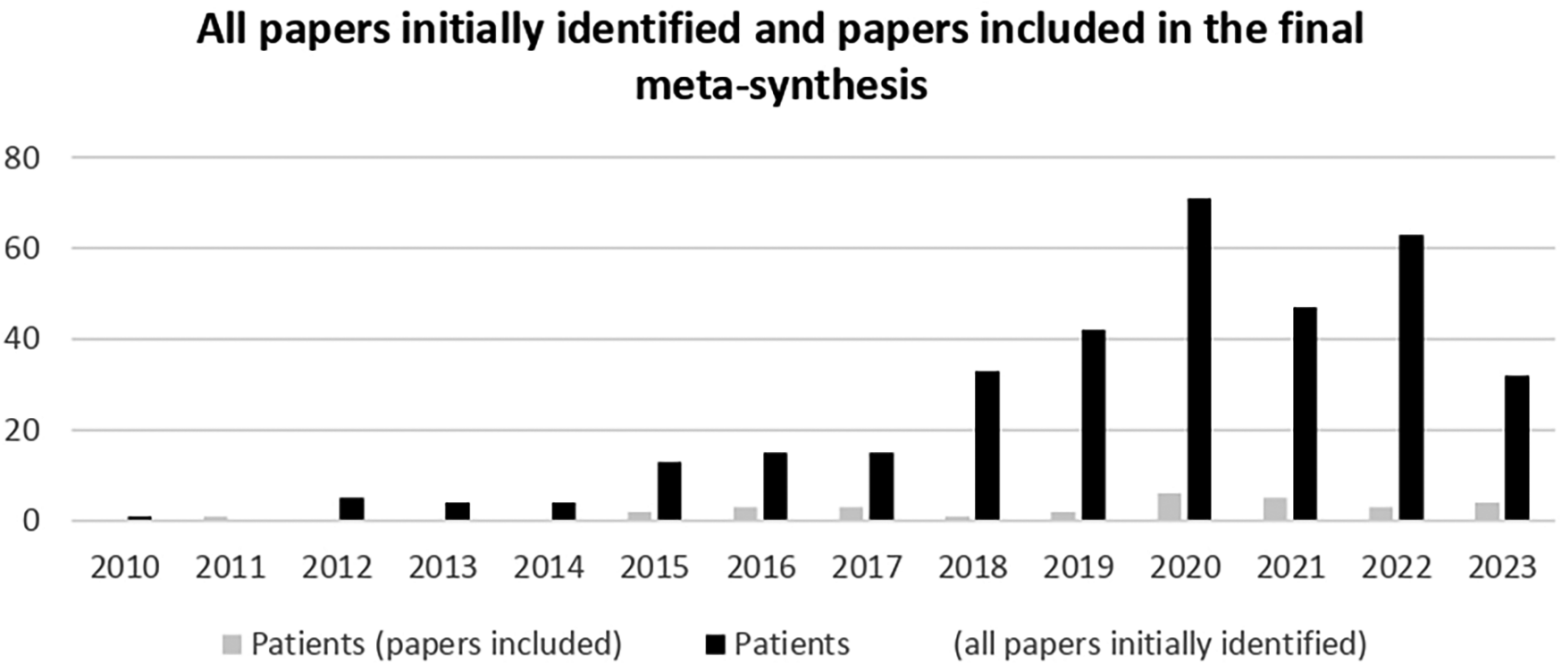
Comparison of initially and included papers by years of publication (2010-2023).

The same was true for the papers included in the final meta-synthesis (Figure 4). To examine the evolution of digital health research, we analyzed both the initially identified papers (*n* = 345) and the final included papers (*n* = 28) by year of publication. Figure 4 illustrates this distribution, highlighting an increasing trend in related studies since 2015, with a peak in 2020. This surge aligns with the broader expansion of digital health research (Figure 2) and likely reflects the accelerated adoption of digital technologies by patients during the COVID-19 pandemic.

The main authors' affiliations for the papers included in the meta-synthesis included papers from 13 countries, namely, the USA (*n* = 9), Canada (*n* = 4), the U.K. (*n* = 3), Norway (*n* = 2), Brazil (*n* = 1), Spain (*n* = 1), Germany (*n* = 1), France (*n* = 1), Australia (*n* = 1), Pakistan (*n* = 1), Ethiopia (*n* = 2), Poland (*n* = 1) and Portugal (*n* = 1).

The majority of the papers included in the final meta-synthesis were original reports (*n* = 12), followed by reviews (*n* = 10), systematic reviews (*n* = 4), and opinion/case studies (*n* = 2).

With regard to the type of digital health technology used, as shown in Table 3, the majority of the included papers examined mobile health apps (23/28 papers) and electronic health records (21/28 papers), followed by telemedicine (10/28 papers), artificial intelligence, and wireless monitoring technologies (5/28 papers each), and finally blockchain, digital biomarkers in clinical care and biofeedback/digital rehabilitation technology (1/28 papers each). By analyzing this table, we can identify trends in the adoption of these technologies and understand their distribution across the studies. As seen in the table, mobile health apps are the most commonly discussed technology, followed by electronic health systems and artificial intelligence. This prevalence indicates a strong emphasis on digital tools aimed at improving patient engagement and care delivery.

**Table 3:** Types of technology across included studies.

No	Author	Electronic health	Artificial intelligence	Mobile health apps	Tele-medicine	Wireless monitoring technologies	Digital biomarkers in clinical care	Blockchain technology	Biofeedback/digital rehabilitation technology
1	Cheryl Forchuk et al. (15)	Electronic health record		Mobile health apps					
2	Viral N. Shah et al. (16)	Electronic health record		Mobile health apps					
3	Siobhan O’Connor et al. (17)	Electronic health record		Mobile health apps	Tele-medicine				
4	Carolyn Steele Gray et al. (18)			Mobile health apps					
5	Marcin Kautsch et al. (19)	Electronic health record			Tele-medicine				
6	Janessa Griffith et al. (20)			Mobile health apps					
7	Alex Roehrs et al. (14)	Electronic health record							
8	Kathleen Thies et al. (21)	Electronic health record		Mobile health apps					
9	Jake Carrion (22)	Electronic health record		Mobile health apps	Tele-medicine				
10	Gabriel Ruiz Signorelli et al. (23)		ARTIFICIAL INTELLIGENCE	Mobile health apps	Tele-medicine	Wireless monitoring technologies			Biofeedback digital rehabilitation technology
11	Ryan Shaw et al. (24)	Electronic health record		Mobile health apps		Wireless monitoring technologies			
12	Polina Durneva et al. (25)	Electronic health record			Tele-medicine			Blockchain technology	
13	Miroslav Muzny et al. (26)	Electronic health record		Mobile health apps		Wireless monitoring technologies			
14	Sven Kernebeck et al. (27)	Electronic health record		Mobile health apps	Tele-medicine		Digital biomarkers in clinical care		
15	Oyungerel Byambasuren et al. (28)			Mobile health apps		Wireless monitoring technologies			
16	Ann-Chatrin Linqvist Leonardsen et al. (29)				Tele-medicine				
17	Sundar Jagannath et al. (30)	Electronic health record		Mobile health apps	Tele-medicine				
18	Alice Sarradon-Eck et al. (31)			Mobile health apps					
19	Tibor P. Palfai et al. (32)			Mobile health apps					
20	Binyam Tilahun et al. (33)	Electronic health record	ARTIFICIAL INTELLIGENCE	Mobile health apps	Tele-medicine				
21	Kathryn Hawk et al. (34)	Electronic health record		Mobile health apps					
22	Cláudia Pernencar et al. (35)	Electronic health record	ARTIFICIAL INTELLIGENCE	Mobile health apps					
23	Adriana Guzman et al. (36)	Electronic health record		Mobile health apps					
24	Yvonne Bombard et al. (37)	Electronic health record	ARTIFICIAL INTELLIGENCE						
25	Ullah Muneeb et al. (38)	Electronic health record	ARTIFICIAL INTELLIGENCE	Mobile health apps		Wireless monitoring technologies			
26	Getachew Emnet et al. (39)	Electronic health record		Mobile health apps	Tele-medicine				
27	Annabelle Painter et al. (40)	Electronic health record		Mobile health apps					
28	Cathy Shields et al. (41)	Electronic health record		Mobile health apps					

To provide a detailed overview of the studies included in the final meta-synthesis (*n* = 28), we compiled their key characteristics in Supplementary Table 2. This table outlines essential information such as study design, population, digital health interventions, and key findings, offering deeper insights into the scope and focus of the included research. This structured summary enhances transparency and facilitates a comprehensive understanding of the evidence base.

### 3.4 Categorization of reported gaps and challenges in the included papers

Using the prespecified terms to denote gaps and challenges (see Supplementary Table 3), an initial categorization of reported outcomes was performed. It highlights key thematic areas and associated technological considerations. This classification enables a structured analysis of the primary obstacles faced in adopting these technologies, supporting a targeted approach to addressing these issues in future research and policy development.

To further refine the analysis, the reported findings were further grouped into seven new conceptual categories (see Supplementary Table 4): (1) ethical issues, (2) educational/training issues, (3) usability issues, (4) communication issues, (5) functionality/structural issues, (6) technical issues, and (7) access issues. These categories offer a systematic way to interpret and address the barriers identified across the studies. By organizing the findings in this way, we aim to facilitate a clearer understanding of the most pressing challenges and inform targeted solutions for future research and policy development. The most populated category was related to ethical issues, reported in 19 papers, and the least common was access issues, reported in only 8 papers.

A final step included further grouping into three main overarching pillars that provided the foundation for the meta-synthesis and the formulation of final recommendations. The study findings from the existing literature were evaluated using thematic content analysis. Our narrative synthesis was then structured around the categories derived from the included study results or outcomes. These categories were: (A) digital literacy (encompasses issues related to user knowledge, skills, and confidence in engaging with digital health tools), (B) functionality/usability (captures concerns regarding system design, accessibility, and ease of use), and (C) trust (reflects factors such as data security, ethical considerations, and confidence in digital solutions among stakeholders) (see Supplementary Table 5). These pillars emerged as central themes that encapsulate the key challenges and considerations for digital health technology adoption. Digital Literacy. Functionality/Usability. Trust. By structuring our synthesis around these categories, we aim to offer a clearer framework for addressing critical barriers and facilitating more effective implementation of digital health technologies.

The results showed that digital health literacy deficits (17, 20, 27, 30, 39) were the most commonly identified deficits. Such deficits included a lack of awareness of the existence of health technology due to lack of promotion (17), as well as English literacy issues for patients whose first language was not English (17, 33). The most common perceived challenges were fear and frustration as a result of not fully understanding the technologies used (15, 17, 32, 33), along with low confidence in the patient's ability to interpret health data in electronic health records (EHRs) and mHealth apps, which resulted in increased anxiety and concern about incorrect self-diagnosis and taking inappropriate steps to seek care (33, 40).

With regard to functionality/usability, the most common perceived gap was the lack of internet or smartphone access (16, 17, 21, 27, 30, 32–34) reported in 8 papers, while the most common perceived challenge was patients' inability to use digital technology (17, 30, 38–40) due to poor technological awareness and subsequent feelings of incapability (17, 30), followed by age criteria (17, 30), mainly referring to older patients (16, 38, 40), which were reported in 5 papers.

The most common gap related to trust was related to issues related to the privacy and security of personal information, which was reported in 10 papers (14, 16, 17, 21–23, 25, 27, 30, 32), thus revealing the crucial need for relevant regulatory frameworks (38). The lack of control over one's own data (25) has highlighted the need to negotiate regulatory issues surrounding licensing (30). The most common perceived challenge was the user's resistance to change (19, 25, 38), which was reported in 3 papers. Concerns about the impact of digital health services on the patient-physician relationship (18, 40) have created concerns about missing human connections with doctors. Finally, reliance on the user's input (14, 22, 38) was also reported in 3 papers, enhancing the potential for misinterpretation.

## 4 Discussion

This study aimed to identify and map the perceived gaps and challenges associated with digital health technology use among patients in the published literature and to perform a meta-synthesis. A total of 28 papers were included in the final meta-synthesis, ranging from 2015 to the end of 2023, with most of the publications originating from developed countries. A total of 99 findings were reported from these papers; 25 were identified as gaps, and 74 were identified as challenges. These issues were further grouped into seven thematic categories: (1) ethical issues, (2) educational/training issues, (3) usability issues, (4) communication issues, (5) functionality/structural issues, (6) technical issues, and (7) access issues. The most commonly reported gap was the issue of privacy and security of personal information, while the most frequently reported challenge was a lack of familiarity with digital health technologies, often due to age-related factors among older patients.

All the findings were thematically abstracted and ultimately conceptually synthesized into three main pillars/problem dimensions—(A) digital literacy, (B) functionality/usability, and (C) trust—which should be considered for future research and policy recommendations. Trust was set as a separate category to highlight the importance it may have in user preference/usability.

The first pillar/problem dimension can be identified around digital health literacy deficits (17, 20, 27, 37), some of which can be traced back to a generational difference in overall digital propensity (28). Patients may not even be aware of the existence of health technology (17); even when they do, they may be inexperienced with the use of such technology (15, 32) or have not developed either the digital (41) or the technical skills (17) necessary to interact with technology effectively. In addition to the technology literacy barrier (38), patients also report English literacy issues (17, 33) since English may not be their first language. This situation can reportedly cause feelings of fear and frustration (15, 32); increased anxiety (33); worry; and concern about possible incorrect self-diagnosis or taking inappropriate steps to seek care (40). These feelings may lead to low confidence in the patient's ability to interpret health data (40) and to a loss of motivation to improve their health through electronic data (17).

The second pillar/problem dimension refers to technical/technological/functional deficiencies during the use and access of digital technology by patients. Even though the World Health Organization (WHO) emphasizes that digital health is essential for achieving universal health coverage, as “it extends the scope, transparency, and accessibility of health services and health information, widening the population base capable of accessing the available health services and offering innovation and efficiency gains in the provision of health care” (42), patients still deal with access issues, including lack of computer or mobile equipment (17) and lack of internet access (16, 17, 27, 30) or system connectivity errors (18, 33) and limitations in good quality of the internet connection (19). All of the above may result in ongoing concern and frustration for patients. Moreover, although a growing body of literature suggests that the adoption of digital health technologies is associated with higher-quality care, usability concerns (38) can cause patients to feel dissatisfied with their overall experience with digital health technology. A key factor in the inability to use technology was poor app usability (28), leading to user abandonment (23). One reason users disengage from apps is the time commitment (21, 28), while others include patients' dissatisfaction with the app's manual input process (23), the need to remember passwords (15, 34), logging in every time, and waiting for the loading process (15), which are burdensome and reduce users' interest. A feeling of frustration was reported by patients about other technical issues regarding functionality and appearance, including but not limited to a perceived stigma and embarrassment when they had chosen to wear a device, while the frequent need to recharge was also a disadvantage (29). Age (16, 27, 38, 40), language (16, 38, 40), and cultural barriers to effective communication (38) affect users' compliance (38) and increase concerns about inequity in the use and access of digital health services (33, 40).

The third pillar/problem dimension refers to ethical deficiencies, which further result in a lack of trust in technology. Issues of privacy and security, confidentiality, and integrity of personal health information (14, 17, 21–23, 25, 27, 30, 32) were key concerns as reported by patients. In particular, concerns about the potential misuse of data, including risks around cybersecurity breaches and commercial end-use (16, 40), along with concerns about data being shared with governmental bodies (16, 40), were reported. Moreover, perceived issues regarding data repository ownership (14), secondary use of personal data (24), and lack of control over data (25) all highlight patients' need to develop regulatory frameworks (38). Concerning mobile apps, the majority of users may not even understand all ethical issues arising from their use (35) since no seal or certification exists that would make it easier for the end-user to understand which products use a high industry-standard level of security and are safe to use (27). On the other hand, users' resistance to change (19, 38) and subsequently to new technologies (25, 32) remain significant barriers that governments and developers need to take into account. Patients report a need to trust and rely on technology and that all information comes from a trustworthy source (23) while also expressing fears of being linked to their digital identity (25) and worries about identity fraud (17). Furthermore, the impact of digital technology on the patient-physician relationship is also an important parameter. The lack of human contact when interacting with technology (30) and the possible development of abusive or threatening behaviour (17) during virtual health meetings can lead patients to feel disconnected, preventing them from being engaged and enrolled. Trusting advice from a virtual health professional without input from a qualified doctor or nurse (17) is an additional issue. The sense of isolation and alienation, as digital tools replace in-person consultation, and the lack of interpersonal reassurance may lead to a perceived reduction in holistic care (40), while the risk of addiction to technology resulting in distortions of social relationships and cognitive perceptions toward critical thinking was also mentioned (31). Finally, socioeconomic parameters seem to affect the adoption of digital technology, as patients were discouraged from thinking that real-life doctors might be affordable only for those with adequate insurance or financial resources, while others would be predominantly treated by avatars or telemedical consultants (27).

Although we used a bottoms-up thematic analysis to group first in larger and then in more concise categories the identified gaps and challenges from the included literature, these fit well with the recently published socio-technical framework, proposed by Jacob et al. (43) further supporting our categorization, especially with regard to functionality and usability, and further technical and cultural requirements, data protection/safety and regulatory compliance. They suggest a sociotechnical framework assessing patient-facing eHealth tools to equip decision-makers, emphasizing both social and technical aspects as interdependent parts of a complex system (44), going beyond evaluating individual technologies and taking into account their intended context, categorizing criteria as foundational and contextual. In addition, another recent publication [Cordeiro (45)] reporting on the ethical, legal, and social implications of the adoption of digital technologies, further supports the role of trust as a fundamental value, in agreement with our thematic categorization of *Trust* as an independent pillar/problem dimension.

While this study highlights key ethical concerns such as privacy frameworks, trust, and data commodification risks, a more in-depth analysis of mechanisms to enhance trust is required. Future research should focus on proposing concrete strategies and guidelines for developers, policymakers, and stakeholders. These could include implementing transparent consent processes that ensure users have clear and informed choices regarding their data, establishing robust data governance frameworks that prioritize security and ethical data use, and designing methodologies to foster user trust in digital health technologies. Incorporating these strategies would not only enhance the ethical discourse but also provide practical recommendations that contribute to responsible and equitable digital health adoption.

### 4.1 Interoperability and data integration challenges

One of the most significant challenges in digital health technology is interoperability—the ability of different health systems, platforms, and devices to exchange and use patient data seamlessly. The lack of standardized frameworks for data sharing across healthcare providers and institutions often results in fragmented patient records, impeding coordinated care.

Several studies in our review highlight interoperability as a key barrier to effective digital health implementation. Differences in electronic health record (EHR) systems, proprietary software restrictions, and varying data formats limit the ability to integrate patient information across multiple healthcare providers. This lack of integration can lead to inefficiencies, misdiagnoses, and gaps in continuity of care, ultimately affecting patient outcomes. Additionally, concerns around data privacy and security complicate interoperability efforts. Healthcare organizations must balance the need for data accessibility with stringent regulatory requirements, such as GDPR and HIPAA, which impose restrictions on cross-institutional data sharing. Without clear policies and technical solutions, patient data remains siloed, restricting its full potential in enhancing healthcare delivery.

To address these challenges, policymakers and technology developers should prioritize the development of standardized data exchange protocols, improved interoperability frameworks, and secure, scalable digital health infrastructures. Future research should explore best practices for achieving interoperability and investigate patient-centered solutions that enhance data fluidity while preserving privacy and security.

### 4.2 Practical implications for digital health technology development and policy

The findings highlight the need for digital health technologies to be adaptable to diverse geographic and demographic contexts. Studies predominantly from high-income regions may introduce biases, overlooking the challenges faced by populations in LMICs or rural areas. For technology development, solutions must be tailored to varying infrastructures, with low-cost, accessible options for underserved regions. Policy should focus on promoting equitable access, supporting infrastructure improvements, and enhancing digital literacy, particularly in low-resource areas. Acknowledging these biases is crucial, and future research should include more diverse study populations to ensure that findings are applicable globally.

### 4.3 Considerations for low- and middle-income countries

As most studies in this review are from the USA and Europe, the findings may not be directly applicable to low- and middle-income countries (LMICs) due to differences in healthcare infrastructure, technological access, and resources. In LMICs, barriers such as limited internet access, lower digital literacy, and healthcare system challenges could impact the adoption and effectiveness of digital health tools. However, digital health technologies also offer opportunities to address unique challenges in these regions, such as improving access to care in remote areas and providing cost-effective solutions. Future research should focus on adapting these technologies to LMIC contexts and evaluating their impact on healthcare delivery in these settings.

### 4.4 Impact of digital health technology on health outcomes

Several studies included in this review highlight the impact of digital health technologies on health outcomes. Evidence suggests that mobile health apps, telemedicine, and electronic health records contribute to improved disease management, enhanced patient engagement, and better clinical outcomes. For instance, remote monitoring and telehealth services have been shown to enhance the management of chronic conditions such as diabetes, cardiovascular diseases, and mental health disorders by enabling continuous tracking and real-time patient-provider interactions.

Digital interventions facilitate timely medical advice, reducing hospital readmissions and improving adherence to treatment plans. Additionally, mobile health applications empower patients by increasing self-management capabilities, promoting health literacy, and encouraging lifestyle modifications that lead to better long-term health outcomes.

Despite these benefits, some studies highlight concerns related to disparities in access, usability issues, and data security risks, which may hinder the widespread adoption of digital health solutions. Ensuring equitable access and addressing digital literacy challenges remain critical factors in maximizing the potential of digital health technology to improve patient outcomes. Future research should further explore region-specific implications, particularly in low- and middle-income countries, to understand the broader impact of digital health tools on healthcare delivery and patient well-being.

### 4.5 Regulatory, quality, and certification considerations for digital health technologies

Given the increasing reliance on digital health technologies, ensuring their quality, safety, and efficacy is crucial to fostering trust among users. Digital health technologies, including medical devices and digital therapeutics, require robust regulatory frameworks to ensure compliance with safety standards and clinical effectiveness.

Several regulatory bodies, such as the U.S. Food and Drug Administration (FDA), the European Medicines Agency (EMA), and national health authorities, oversee the approval and certification of digital health technologies. These regulations focus on aspects such as data security, clinical validation, software reliability, and interoperability with existing healthcare systems.

Quality certification mechanisms, such as the Digital Health Certification by the International Organization for Standardization (ISO) and the Health IT Certification Program, establish best practices for ensuring the safety and effectiveness of digital health applications. Implementing standardized regulatory pathways can help mitigate risks associated with data privacy, cybersecurity threats, and disparities in access, ultimately enhancing patient confidence in digital health solutions.

Furthermore, increased transparency in data governance and the ethical use of patient information are essential for maintaining public trust. Future advancements in regulatory policies should focus on balancing innovation with rigorous safety assessments, ensuring that digital health technologies contribute positively to healthcare outcomes while safeguarding patient rights and security.

### 4.6 Consideration of rapid technological development

The meta-synthesis covers a 10-year period during which rapid advancements in digital health took place. While the review captures key trends and challenges in digital health adoption, it may not fully reflect the most recent developments, particularly in artificial intelligence (AI) and its integration into medical devices. AI-powered digital therapeutics, predictive analytics, and machine learning-driven diagnostics have gained significant momentum in recent years, presenting both opportunities and new challenges for patient care.

As AI-driven medical devices and digital solutions continue to evolve, future research should focus on assessing their impact on patient outcomes, regulatory compliance, and ethical considerations. Additionally, interoperability and standardization remain critical issues as AI-based solutions require seamless integration with existing healthcare infrastructures. Addressing these emerging developments will be essential in ensuring that digital health technologies remain both effective and trustworthy in modern healthcare ecosystems.

There is no doubt that digital health technology has transformed the healthcare landscape, enhancing patients' empowerment; however, it is crucial to consider the identified gaps and challenges in technology use as perceived by its users, especially with regard to the disruption of human rights. The issues reported highlight the possible intensification of social health inequalities, the exploitation and commodification of personal health information, the decrease in patient autonomy, the doubted value of patient-generated data due to potential increased subjectivity, the dehumanization/distortion of the patient-doctor relationship, and the addiction to technology.

### 4.7 Addressing dehumanization in the patient-doctor relationship

Digital health technologies, while beneficial, may inadvertently reduce the human connection in healthcare by stripping away non-verbal cues and emotional engagement. This can lead to feelings of alienation for patients. However, when designed thoughtfully, these technologies can complement, rather than replace, human interactions. Video consultations, for example, preserve face-to-face communication and enhance empathy, while digital tools like EHRs can reduce administrative tasks, allowing more time for patient interaction. Studies have shown that digital platforms, when used correctly, can improve communication and patient satisfaction. For instance, research by Madanian et al. (46) highlights how digital tools empower patients and improve engagement. Similarly, Polus et al. (47) found that digital health interventions in chronic care led to better outcomes through more frequent, meaningful interactions. Designing digital health technologies with a focus on user-centered care and supporting, not replacing, human interactions can enhance compassionate healthcare delivery.

Based on the results of this meta-synthesis, the issues highlighted here need to be discussed among all stakeholders and included in the development of relevant digital technologies, which is in agreement with Shaw and Donia's (48) suggested socio-technical system, focusing on social justice for the communities implicated in the development and distribution of digital health technology with the aim of creating a better world for all. Therefore, we propose that a bioethics model for ethically aligned technology acceptance under the scope of human rights is necessary for the digital healthcare ecosystem, satisfying the ethical principles of autonomy, privacy, and equality.

### 4.8 Ethical frameworks for digital health development

To guide the future development of digital health technologies, it is essential to adopt ethical frameworks that address human rights and the challenges identified in this study.

Key considerations include:

1. Human Rights-Based Approach: Technologies must uphold patient rights, such as privacy, informed consent, and equitable access. Safeguards should be in place to prevent misuse of data and protect vulnerable populations.
2. Informed Consent and Autonomy: Ethical frameworks must ensure that patients fully understand how their data is used and have the right to control their participation in digital health tools.
3. Equity and Access: Digital health tools should be accessible to all, particularly underserved groups, with features that bridge the digital divide, such as multilingual support and compatibility with low-cost devices.
4. Human Interaction and Empathy: Ethical guidelines should prioritize maintaining human connection in care. Technology should support, not replace, empathetic communication between patients and providers.
5. Data Protection and Security: Robust data protection measures are essential to maintain trust in digital health solutions. Ethical frameworks must ensure that patient data is securely managed and protected from breaches.

By applying these ethical principles, developers, healthcare providers, and policymakers can create digital health technologies that respect patient rights and enhance healthcare quality.

## 5 Limitations

It is important to note some limitations associated with our study. Most of the included papers originated from the USA and Europe, thus making generalization difficult. This geographical bias restricts the global applicability of our findings, as healthcare systems, cultural attitudes, and technological infrastructures vary significantly across regions. Consequently, insights from non-Western contexts, where different economic, systemic, and cultural factors influence digital health adoption, remain underrepresented. Future research should actively seek to include studies from diverse geographic settings to provide a more comprehensive and globally relevant understanding of digital health challenges and opportunities. Furthermore, language restrictions were used in the search strategy, thus limiting the included publications to those in English. This may have excluded valuable research published in other languages, particularly from regions where English is not the primary language of academic publication. Although we searched the 3 most relevant databases on this topic, we cannot rule out the possibility that including additional databases might have yielded additional relevant publications. We included only published studies and not unpublished or gray literature, which may have resulted in missing some relevant unpublished data. However, we decided to synthesize only published studies as a basic quality indicator of the included data. Only 12 of the 28 included papers were original reports (and of various methodologies), while 14 were reviews (4 of which were systematic), allowing for the possibility of additional relevant gaps and challenges not being reported in the literature due to lack of original studies on the topic. What is more, it is possible that some bioethics and sociological literature on patient use of digital health tools has not been captured by our search terms. This could most likely be due to differences in the terminology used. However, even though it is possible that individual issues may have been overlooked, it is unlikely that they would fall outside of the three final pillars/problem dimensions.

Finally, another limitation is the manuscript's heavy focus on barriers and gaps, which, while important, risks creating an overly critical narrative. However, several successful implementations of digital health technologies demonstrate how these challenges can be overcome. For example, Estonia's national health information system has effectively integrated electronic health records (EHRs) to enhance patient access and interoperability, ensuring seamless data exchange while maintaining robust privacy protections (49). Similarly, the UK's NHS digital transformation initiatives, such as the NHS App, have successfully facilitated patient engagement, appointment scheduling, and digital prescriptions, improving accessibility and efficiency (50). Furthermore, initiatives like Rwanda's Babyl digital health platform have shown how telemedicine can address healthcare access challenges in resource-limited settings (51). Highlighting such best practices provides a more balanced and constructive perspective on digital health adoption.

## 6 Conclusion

Digital health technology has the potential to revolutionize healthcare delivery and provide better care to everyone. Digitally enhanced patient empowerment can increase patient independence. Nonetheless, relevant gaps and challenges in technology use by its intended users need to be considered, especially issues pertaining to human rights, as highlighted by our findings. In this context, it is crucial to note that all three identified pillars contain issues that can be traced to a disruption of human rights, with issues of trust remaining a major concern. An analysis of the above leads to issues related to the intensification of social health inequalities, the exploitation and commodification of personal health information, a decrease in patient autonomy, the doubted value of patient-generated data due to potential increased subjectivity, the dehumanization/distortion of the patient-doctor relationship, and addiction to technology. Such issues need to be discussed among all stakeholders and included in the development of relevant digital technologies. Therefore, we propose that a bioethics model for ethically aligned technology acceptance under the scope of human rights is necessary in the digital healthcare ecosystem. Such a model would include issues of autonomy, privacy, and equality, as highlighted in the present meta-synthesis.

Based on the identified gaps and challenges, providing concise recommendations for stakeholders is crucial. To enhance the impact of this research, we recommend the following actions for key stakeholder groups:

Policy Makers:

• Regulate and Support: Establish clear regulations for integrating digital health technologies while preserving human interactions. Promote funding for research on the long-term effects of digital health.
• Promote Digital Literacy: Support initiatives to improve digital literacy for both healthcare providers and patients, especially among underserved groups.

Healthcare Institutions:

• Train Providers: Offer ongoing training for healthcare providers on using digital tools in ways that enhance empathy and communication.
• Adopt Patient-Centered Technologies: Prioritize technologies that improve patient care while maintaining human connection.
• Encourage Collaboration: Foster collaborative care models that integrate digital health tools into multidisciplinary teams.

Technological Developers:

• Design User-Friendly Tools: Focus on creating intuitive, accessible digital solutions that complement human interaction, including video consultations and real-time communication.
• Ensure Privacy and Security: Prioritize data protection and security to maintain trust in digital health tools.
• Promote Interoperability: Ensure seamless integration with existing healthcare systems to support smooth transitions and continuity of care.

By addressing these areas, stakeholders can help ensure that digital health technologies enhance both the efficiency and humanity of healthcare delivery.

## Data Availability

The original contributions presented in the study are included in the article/Supplementary Material, further inquiries can be directed to the corresponding author.

## References

[1] Awad A Trenfield SJ Pollard TD Ong JJ Elbadawi M McCoubrey LE Connected healthcare: improving patient care using digital health technologies. Adv Drug Deliv Rev. (2021) 178:113958. 10.1016/j.addr.2021.113958 34478781

[2] Ibrahim MS Mohamed Yusoff H Abu Bakar YI Thwe Aung MM Abas MI Ramli RA. Digital health for quality healthcare: a systematic mapping of review studies. Digit Health. (2022) 8:20552076221085810. 10.1177/20552076221085810 35340904 PMC8943311

[3] Marcolino MS Oliveira JAQ D'Agostino M Ribeiro AL Alkmim MBM Novillo-Ortiz D. The impact of mHealth interventions: systematic review of systematic reviews. JMIR Mhealth Uhealth. (2018) 6:e8873. 10.2196/mhealth.8873 29343463 PMC5792697

[4] Arden NS Fisher AC Tyner K Yu LX Lee SL Kopcha M. NC-ND license industry 4.0 for pharmaceutical manufacturing: preparing for the smart factories of the future. Int J Pharm. (2021) 602:120554. 10.1016/j.ijpharm.2021.120554 33794326

[5] Brørs G Norman CD Norekvål TM. Accelerated importance of eHealth literacy in the COVID-19 outbreak and beyond. Eur J Cardiovasc Nurs. (2020) 19(6):458–61. 10.1177/1474515120941307 32667217 PMC7480020

[6] Kumar KPA Pumera M. 3D-printing to mitigate COVID-19 pandemic. Adv Funct Mater. (2021) 31(22):2100450. 10.1002/adfm.202100450 34230824 PMC8250363

[7] Gunasekeran DV Tham YC Ting DSW Tan GSW Wong TY. Digital health during COVID-19: lessons from operationalising new models of care in ophthalmology. Lancet Digit Health. (2021) 3(2):e124–34. 10.1016/S2589-7500(20)30287-9 33509383

[8] Linqvist Leonardsen AC Hardeland C Helgesen AK Grøndahl VA. Patient experiences with technology enabled care across healthcare settings-a systematic review. BMC Health Serv Res. (2020) 20:779. 10.1186/s12913-020-05633-4 32838784 PMC7446109

[9] Moher D Liberati A Tetzlaff J Altman DG Altman D Antes G Preferred reporting items for systematic reviews and meta-analyses: the PRISMA statement. PLoS Med. (2009) 6:e1000097. 10.1371/journal.pmed.1000097 19621072 PMC2707599

[10] Oliver S Sutcliffe K. Describing and analysing studies. In: David G Sandy O James T, editors. An Introduction to Systematic Reviews. London: Sage (2012). p. 135–52. Available at: https://www.scribd.com/document/518926883/Gough-David-Oliver-Sandy-Thomas-James-An-Introduction-to-Systematic-Reviews-2013-SAGE-Publications-Libgen-lc# (Accessed January 10, 2023).

[11] Zheng X Zhang J Ye X Lin X Liu H Qin Z Navigating through motherhood in pregnancy and postpartum periods during the COVID-19 pandemic: a systematic review and qualitative meta-synthesis. J Nurs Manag. (2022) 30(8):3958. 10.1111/jonm.13846 36194367 PMC9874529

[12] Zheng X Qian M Ye X Zhang M Zhan C Li H Implications for long COVID: a systematic review and meta-aggregation of experience of patients diagnosed with COVID-19. J Clin Nurs. (2024) 33(1):40–57. 10.1111/jocn.16537 36253950 PMC9874539

[13] Lockwood C Munn Z Porritt K. Qualitative research synthesis: methodological guidance for systematic reviewers utilizing meta-aggregation. Int J Evid Based Healthc. (2015) 13(3):179–87. 10.1097/XEB.0000000000000062 26262565

[14] Roehrs A Da Costa CA Da Rosa Righi R De Oliveira KSF. Personal health records: a systematic literature review. J Med Internet Res. (2017) 19:e5876. 10.2196/jmir.5876 28062391 PMC5251169

[15] Forchuk C Reiss JP O'Regan T Ethridge P Donelle L Rudnick A. Client perceptions of the mental health engagement network: a qualitative analysis of an electronic personal health record. BMC Psychiatry. (2015) 15(1):1–11. 10.1186/s12888-015-0614-7 26467210 PMC4606496

[16] Shah VN Garg SK. Managing diabetes in the digital age. *Clin Diabetes Endocrinol*. (2015) 1(1). Available at: https://pubmed.ncbi.nlm.nih.gov/28702234/10.1186/s40842-015-0016-2 PMC5471958 28702234

[17] O'Connor S Hanlon P O'Donnell CA Garcia S Glanville J Mair FS. Understanding factors affecting patient and public engagement and recruitment to digital health interventions: a systematic review of qualitative studies. BMC Med Inform Decis Mak. (2016) 16:1–15. 10.1186/s12911-016-0359-3 27630020 PMC5024516

[18] Gray CS Gill A Khan AI Hans PK Kuluski K Cott C. The electronic patient reported outcome tool: testing usability and feasibility of a mobile app and portal to support care for patients with complex chronic disease and disability in primary care settings. JMIR Mhealth Uhealth. (2016) 4(2):e5331. 10.2196/mhealth.5331 27256035 PMC4911509

[19] Kautsch M Lichoń M Matuszak N. Ehealth development in selected EU countries: barriers and opportunities. Int J Integr Care. (2016) 16(6):97. 10.5334/ijic.2645

[20] Griffith J Monkman H. Usability and eHealth literacy evaluation of a mobile health application prototype to track diagnostic imaging examinations. In: Mantas J, Hasman A, Gallos P, Househ MS, editors. Studies in Health Technology and Informatics. Amsterdam: IOS Press BV (2017). p. 150–5. 28186032

[21] Thies K Anderson D Cramer B. Lack of adoption of a mobile app to support patient self-management of diabetes and hypertension in a federally qualified health center: interview analysis of staff and patients in a failed randomized trial. JMIR human Factors. (2017) 4(4):e7709. 10.2196/humanfactors.7709 28974481 PMC5645643

[22] Carrion J. Improving the patient-clinician interface of clinical trials through health informatics technologies. J Med Syst. (2018) 42:1–6. 10.1007/s10916-018-0973-y 29845581

[23] Signorelli GR Lehocki F Fernández MM O'Neill G O'Connor D Brennan L A research roadmap: connected health as an enabler of cancer patient support. J Med Internet Res. (2019) 21:e14360. 10.2196/14360 31663861 PMC6914240

[24] Shaw R Stroo M Fiander C McMillan K. Selecting mobile health technologies for electronic health record integration: case study. J Med Internet Res. (2020) 22(10):e23314. 10.2196/23314 33112248 PMC7657715

[25] Durneva P Cousins K Chen M. The current state of research, challenges, and future research directions of blockchain technology in patient care: systematic review. J Med Internet Res. (2020) 22:e18619. 10.2196/18619 32706668 PMC7399962

[26] Muzny M Henriksen A Giordanengo A Muzik J Grøttland A Blixgård H Wearable sensors with possibilities for data exchange: analyzing status and needs of different actors in mobile health monitoring systems. Int J Med Inf. (2020) 133:104017. 10.1016/j.ijmedinf.2019.104017 31778885

[27] Kernebeck S Busse TS Böttcher MD Weitz J Ehlers J Bork U. Impact of mobile health and medical applications on clinical practice in gastroenterology. World J Gastroenterol. (2020) 26:4182–97. 10.3748/wjg.v26.i29.4182 32848328 PMC7422538

[28] Byambasuren O Beller E Hoffmann T Glasziou P. Barriers to and facilitators of the prescription of mhealth apps in Australian general practice: qualitative study. JMIR Mhealth Uhealth. (2020) 8(7):e17447. 10.2196/17447 32729839 PMC7426799

[29] Leonardsen ACL Hardeland C Helgesen AK Grøndahl VA. Patient experiences with technology enabled care across healthcare settings- a systematic review. BMC Health Serv Res. (2020) 20(1):1–17. 10.1186/s12913-019-4778-6 PMC7446109 32838784

[30] Jagannath S Mikhael J Nadeem O Raje N. Digital health for patients with multiple myeloma: an unmet need. JCO Clin Cancer Inform. (2021) 5(5):1096–105. 10.1200/CCI.20.00145 34735265

[31] Sarradon-Eck A Bouchez T Auroy L Schuers M Darmon D. Attitudes of general practitioners toward prescription of mobile health apps: qualitative study. JMIR Mhealth Uhealth. (2021) 9(3):e21795. 10.2196/21795 33661123 PMC7974757

[32] Palfai TP Kratzer MPL Morone NE Bernstein JA. Integrating patient perspectives in the development of a mobile health intervention to address chronic pain and heavy drinking in primary care: a qualitative study of patients in an urban, safety-net hospital setting. Addict Sci Clin Pract. (2021) 16:20. 10.1186/s13722-021-00230-0 33757584 PMC7988929

[33] Tilahun B Gashu KD Mekonnen ZA Fikadie Endehabtu B Angaw DA. Mapping the role of digital health technologies in prevention and control of COVID-19 pandemic: review of the literature. Yearb Med Inform. (2021) 30(1):26–37. 10.1055/s-0041-1726505 34479378 PMC8416203

[34] Hawk K Malicki C Kinsman J D'onofrio G Taylor A Venkatesh A. Feasibility and acceptability of electronic administration of patient reported outcomes using mHealth platform in emergency department patients with non-medical opioid use. Addict Sci Clin Pract. (2021) 16:66. 10.1186/s13722-021-00276-0 34758881 PMC8579535

[35] Pernencar C Saboia I Dias JC. How far can conversational agents contribute to IBD patient health care—a review of the literature. Front Public Health. (2022) 10:862432. 10.3389/fpubh.2022.862432 35844879 PMC9282671

[36] Guzman A Brown T Liss DT. "It closes the gap when the ball is dropped": patient perspectives of a novel smartphone app for regional care coordination after hospital encounters. Mhealth. (2022) 8:13. 10.21037/mhealth-21-49 35449511 PMC9014227

[37] Bombard Y Ginsburg GS Sturm AC Zhou AY Lemke AA. Digital health-enabled genomics: opportunities and challenges. Am J Hum Genet. (2022) 109(7):1190. 10.1016/j.ajhg.2022.05.001 35803232 PMC9300757

[38] Ullah M Hamayun S Wahab A Khan SU Qayum M Ullah A Smart technologies used as smart tools in the management of cardiovascular disease and their future perspective. Curr Probl Cardiol. (2023) 48(11):101922. 10.1016/j.cpcardiol.2023.101922 37437703

[39] Getachew E Adebeta T Muzazu SGY Charlie L Said B Tesfahunei HA Digital health in the era of COVID-19: reshaping the next generation of healthcare. Front Public Health. (2023) 11:942703. 10.3389/fpubh.2023.942703 36875401 PMC9976934

[40] Painter A van Dael J Neves AL Bachtiger P O'Brien N Gardner C Identifying benefits and concerns with using digital health services during COVID-19: evidence from a hospital-based patient survey. Health Informatics J. (2023) 29(4):14604582231217339. 10.1177/14604582231217339 38011503

[41] Shields C Conway NT Allardice B Wake DJ Cunningham SG. Continuing the quality improvement of an electronic personal health record and interactive website for people with diabetes in Scotland (my diabetes my way). Diabetic Med. (2023) 40(7):e15085. 10.1111/dme.15085 36924001

[42] Health Organization Regional Office for Europe W. From innovation to implementation eHealth in the WHO European Region (2016). Available at: http://www.euro.who.int/en/ehealth (Accessed February 20, 2025).

[43] Jacob C Lindeque J Müller R Klein A Metcalfe T Connolly SL A sociotechnical framework to assess patient-facing eHealth tools: results of a modified delphi process. NPJ Digital Medicine. (2023) 6(1):1–15. 10.1038/s41746-023-00982-w 38102323 PMC10724255

[44] Mir R Jain S, editors. The Routledge Companion to Qualitative Research in Organization Studies. 1st ed. New York: Routledge (2017). p. 1–514. 10.4324/9781315686103

[45] Cordeiro JV. Digital technologies and data science as health enablers: an outline of appealing promises and compelling ethical, legal, and social challenges. Front Med (Lausanne). (2021) 8:647897. 10.3389/fmed.2021.647897 34307394 PMC8295525

[46] Madanian S Nakarada-Kordic I Reay S Chetty T. Patients’ perspectives on digital health tools. PEC Innovation. (2023) 2:100171. 10.1016/j.pecinn.2023.100171 37384154 PMC10294099

[47] Polus M Keikhosrokiani P Korhonen O Behutiye W Isomursu M. Impact of digital interventions on the treatment burden of patients with chronic conditions: protocol for a systematic review. JMIR Res Protoc. (2024) 13:e54833. 10.2196/54833 38652531 PMC11077406

[48] Shaw JA Donia J. The sociotechnical ethics of digital health: a critique and extension of approaches from bioethics. Front Digit Health. (2021) 3:127. 10.3389/fdgth.2021.725088 34713196 PMC8521799

[49] Overview of the national laws on electronic health records in the EU Member States and their interaction with the provision of cross-border eHealth services Overview of the national laws on electronic health records in the EU Member States National Report for the Republic of Estonia (2014). Available at: http://pub.e-tervis.ee/ (Accessed February 20, 2025).

[50] Sukriti KC Tewolde S Laverty AA Costelloe C Papoutsi C Reidy C Uptake and adoption of the NHS app in England: an observational study. Br J Gen Pract. (2023) 73(737):e932. 10.3399/BJGP.2022.0150 37783512 PMC10562999

[51] Humuza J. Evaluation of Integrated Digital Primary Health Care in Rwanda. Rwanda: AEA RCT Registry, The American Economic Association’s registry for randomized controlled trials (2024). Available at: https://www.socialscienceregistry.org/trials/9823

