# Supplementary material for: The gaps and challenges in digital health technology use as perceived by patients: a scoping review and narrative meta-synthesis
Source: Front Digit Health. 2025 Mar 27;7:1474956. doi: 10.3389/fdgth.2025.1474956 (PMC11983460; doi:10.3389/fdgth.2025.1474956)
Supplement: Supplementary file 3 [file Table3.docx]

**Supplemental Table 3.** Categorization of Reported Outcomes Based on Identified Gaps and Challenges.

| **GAPS** | **CHALLENGES** | |
| --- | --- | --- |
| LACK OF INTERNET OR SMARTPHONE ACCESS | SELECTING THE RIGHT DEVICES FROM THE INCREASING NUMBER OF DIGITAL DEVICES ON THE MARKET | FINDING THE RIGHT APP AT THE RIGHT TIME WHEN PATIENTS DOWNLOADING A MOBILE APP |
| HEALTH & TECHNOLOGY LITERACY DEFICITS/DIGITAL HEALTH LITERACY & LIFE COACHING | MANUAL LOGGING & REGISTRATION OF DIFFERENT OBSERVATIONS & MEASUREMENTS | THE MAJORITY OF USERS DO NOT UNDERSTAND THE ETHICAL ISSUES ASSOCIATED WITH MOBILE APPS WHICH DO NOT OFFER THE RIGHT TO PRIVACY |
| LACK OF HUMAN CONTACT MAKES PEOPLE FEEL DISCONNECTED (TELEMEDICINE) | LACK OF LONG-TERM ENGAGEMENT/USER ABANDONMENT | PUSH NOTIFICATIONS WERE RECEIVED TOO SLOWLY OR TOO OFTEN |
| LACK OF ACCEPTANCE AS A VIABLE OPTION TO MANAGE THEIR CARE WHEN BEING TREATED | THE POTENTIAL FOR PERSONALIZATION/FREQUENT MANUAL INPUT FROM THE USER PERCEIVED AS BURDENSOME & DECREASES INTEREST | THE APP'S GRAPHIC DESIGN LOOKED OUTDATED |
| LACK OF MOTIVATION TO UNDERSTAND & IMPROVE THEIR HEALTH THROUGH ELECTRONIC DATA | USERS CAN BE HESITANT IN SHARING THEIR PERSONAL INFORMATION | SOME ASPECTS OF THE APP WERE TOO WORDY OR LENGTHY |
| LACK OF TECHNICAL SKILLS | INAPPLICABILITY OF REMOTE MONITORING FOR ACUTELY ILL PATIENTS (TELEMEDICINE) | PRIVACY CONCERNS AS A BARRIER TO ACCEPTABILITY SINCE VERY FEW WERE HESITANT ABOUT INFORMATION SECURITY OR ENABLING LOCATION TRACKING |
| LACK OF COMPUTER OR MOBILE EQUIPMENT | INABILITY TO USE DIGITAL HEALTH TECHNOLOGIES/POOR AWARENESS OF TECHNOLOGY/FEELING INCAPABLE OF USING THE TECHNOLOGY (COMPUTERS OR MOBILE DEVICES) | THE ABILITY TO PROVIDE PERSONALIZED FEEDBACK TAILORED TO THEIR PREFERENCES & CHARACTERISTICS |
| LACK OF AWARENESS OF THE EXISTENCE OF HEALTH TECHNOLOGY DUE TO NO WIDE PROMOTION | SEEING NO VALUE IN DHI OFFERED | INCORPORATING PATIENT PROFILES UP FRONT CAN MAKE THE USER EXPERIENCE MORE PATIENT-CENTERED |
| LACK OF CLINICAL ENDORSEMENT | DHI WAS A CONSTANT REMINDER OF THEIR FAILURE TO MEET HEALTHY GOALS & WAS THOUGHT TO BE DISCOURAGING | BUILDING IN CONVERSATIONAL ENTITIES, LIKE CHATBOTS, TO ANSWER QUESTIONS IN REAL TIME CAN BE USED TO SIMULATE A MORE INTERACTIVE USER EXPERIENCE |
| LACK THE NUANCES FOR HUMAN INTERACTION | TECHNOLOGY WAS VIEWED AS POTENTIALLY DISRUPTIVE OR PURELY FOR ENTERTAINMENT PURPOSES & NOT FOR HEALTHCARE NEEDS | FEARS & FRUSTRATIONS AS A RESULT OF NOT FULLY UNDERSTANDING THE TECHNOLOGIES |
| NEED FOR TAILORED EXPERIENCES | PROBLEMS WITH ENGLISH LITERACY SINCE NOT FIRST LANGUAGE | LITTLE TIME OR ENTHUSIASM FOR ENGAGING WITH DHIs/PERSONAL LIFE & VALUES |
| NEED FOR FURTHER DECISION SUPPORT STRUCTURE/SOME WANTED TO SPEAK WITH A CLINICIAN TO CHECK THEIR DECISIONS | INABILITY TO ACCESS AFFORDABLE TECHNOLOGIES DUE TO PROHIBITIVE COSTS INVOLVED | CONCERNS ABOUT SECONDARY USE OF PERSONAL DATA, AS THE USER IS LOCKED WITHIN A LIMITED-OPTIONS ECOSYSTEM OF DEVICE MANUFACTURERS |
| NEED TO NEGOTIATE REGULATORY ISSUES SURROUNDING LICENSING | DIFFICULTIES IN UNDERSTANDING THE RECRUITMENT MESSAGE | CHANGE IN PATIENT-PHYSICIAN RELATIONSHIP |
| PATIENTS NEED TO FEEL THAT THEY CAN RELY ON THE APP/PRIVACY & SECURITY OF PERSONAL INFORMATION | ABUSIVE OR THREATENING BEHAVIOUR DEVELOPED IN VIRTUAL RELATIONSHIPS PREVENTED FROM ENGAGING & ENROLLING | NO SEAL OR CERTIFICATION THAT MAKES IT EASY FOR THE END-USER TO UNDERSTAND WHICH PRODUCTS USE HIGH INDUSTRY-STANDARD LEVELS OF SECURITY & ARE SAFE TO USE |
| PATIENTS NEED TO BE AWARE THAT THE INFORMATION IS COMING FROM A TRUSTWORTHY SOURCE | THE QUALITY OF HEALTH INFORMATION ACCESSED ONLINE WAS THOUGHT UNRELIABLE & THE POTENTIAL FOR IDENTITY FRAUD MAKE IT DIFFICULT FOR SOME TO TRUST ADVICE FROM VIRTUAL HEALTHCARE PROFESSIONALS | RISK THAT REAL-LIFE DOCTORS WILL ONLY BE AFFORDABLE FOR PATIENTS WITH ADEQUATE INSURANCE OR FINANCIAL RESOURCES WHILE OTHERS WILL BE PREDOMINANTLY TREATED BY AVATARS OR TELEMEDICAL CONSULTANTS |
| NEED FOR EVIDENCE OF APP EFFECTIVENESS | THE USABILITY OF DHI WAS FEATURED UNDER QUALITY AS SOME FELT THEY WOULD NOT SIGN UP IF IT WAS SLOW OR CUMBERSOME TO REGISTER OR USE IT | PERSONALIZATION/USABILITY/FAMILIARITY/COMFORT |
| LACK OF EXPERIENCE WITH SIMILAR TECHNOLOGIES CREATED WORRIES/FEAR OF DOING SOMETHING WRONG OR GOING OVER DATA ALLOWANCES | OLDER PATIENTS/AGE CRITERIA | MANAGE MEDICATIONS |
| LACK OF CONTROL OVER DATA | POOR USABILITY OF APPS | PATIENT GENERATED DATA |
| AUTHORIZATION & ACCESS CONTROL TECHNOLOGIES | LOSS OF FINANCIAL INFORMATION | SECURE TRANSPORT PROTOCOL |
| PATIENTS NEED TO BE MOTIVATED TO CONSISTENTLY USE THESE TECHNOLOGIES, ADHERE TO MONITORING PROTOCOLS AND ACTIVELY PARTICIPATE IN THEIR OWN CARE. | TIME COMMITMENT TO HEALTH APPS | DATA REPOSITORY OWNERSHIP |
| NEED FOR REGULATORY FRAMEWORKS. | PARTICIPANTS WERE DISSATISFIED WITH THE NEED TO LOG IN EVERY TIME & WAIT TO THE LOADING PROCESS | CONFIDENTIALITY & INTEGRITY |
| NEED FOR INDIVIDUAL CULTURALLY TAILORING OF THE PROGRAM, ADDRESSING SUPPORT MECHANISMS AND IMPROVE SITE ACCESSIBILITY/ENHANCING THEIR ENGAGEMENT. | THE NEED TO REMEMBER PASSWORDS/FORGOTTEN EMAILS AND PASSWORDS, MULTI-STEP VERIFICATION PROCESSES FOR ACCOUNT | CONCERNS WITH PATIENT-REPORTED MEASURES OF HEALTH DUE TO POTENTIAL INCREASED SUBJECTIVITY |
| LACK OF INTERPERSONAL REASSURANCE | PARTICIPANTS FOUND FLAWS IN THE PROMPTS & REMINDER SYSTEM | INDIVIDUALS MAY BE HESITANT TO DIVULGE HEALTH RELATED INFO, BECAUSE TEXT-MESSAGES BETWEEN PATIENT & PROVIDERS ARE NOT COVERED BY REGULATIONS SET FORTH BY THE HEALTH INSURANCE PORTABILITY & ACCOUNTABILITY ACT |
| MOST PAYERS DO NOT COVER THE COST OF HAVING MEDICAL DEVICES OR APPS DUE TO LACK OF CONCLUSIVE DATA | THE SMARTPHONE WAS CHALLENGING TO USE THAN COMPUTERS | CONCERN ABOUT LINKING PARTICIPANTS TO THEIR DIGITAL IDENTITY |
| INSUFFICIENT SCIENTIFIC EVIDENCES | THE LIMITED BATTERY LIFE OF SMARTPHONES | USER RESISTANCE TO NEW TECHNOLOGIES |
|  | THERE IS AN INCREASING NUMBER OF APPS | ONLY 1 IN 4 REVEALED A QUALITY STANDARD |
|  | TECHNOLOGY LITERACY BARRIER. | LANGUAGE AND CULTURAL BARRIERS IN EFFECTIVE COMMUNICATION AND UNDERSTANDING. |
|  | USER COMPLIANCE AND LONG-TERM ADHERENCE TO WEARABLE'S. | POTENTIAL FOR DISCOMFORT OR SKIN IRRITATION FROM PROLONGED WEAR. |
|  | ETHICAL CONCERNS REGARDING PRIVACY, CONSENT AND POTENTIAL MISUSE OF PATIENT DATA (AI). | RELIANCE ON USER INPUT FOR DATA COLLECTION AND ACCURACY/ POTENTIAL FOR MISINTERPRETATION OR INCORRECT INPUT/RELIANCE ON USER ENGAGEMENT AND MOTIVATION TO CONSISTENTLY USE THE APP. |
|  | ABSENCE OF USER-FRIENDLY FUNCTIONALITIES TO INTERPRET CONSENT DOCUMENTS | LOW CONFIDENCE IN PATIENT'S ABILITY TO INTERPRET HEALTH DATA ON EHRs OR mhealth APPS/RESULTING IN INCREASED ANXIETY AND CONCERN ABOUT INCORRECT SELF-DIAGNOSIS OR TAKING INAPPROPRIATE STEPS TO SEEKING CARE. |
|  | CONCERNS WITH INEQUITY IN ACCESS AND USE OF DIGITAL HEALTH SERVICES/BARRIERS RELATING TO ACCESS STABLE INTERNET OR DIGITAL DEVICES/LANGUAGE BARRIERS/DISABILITIES/LOW DIGITAL LITERACY HINDERING ACCESS AND USE. | CONCERNS WITH COMPROMISED SAFETY/RISK OF MISSED DIAGNOSIS DUE TO THE CLINICIAN'S INABILITY TO PHYSICALLY EXAMINE PATIENTS/ABSENCE OF NON-VERBAL COMMUNICATION WAS PROBLEMATIC. CONCERN ABOUT THE IMPACT OF DIGITAL HEALTH SERVICES ON PATIENT-PHYSICIAN RELATIONSHIP/MISSING HUMAN CONNECTION WITH THE DOCTOR/A PERCEIVED REDUCTION OF HOLISTIC CARE. |
|  | A PERCEIVED STIGMA AND EMBARRASSMENT, AFFECTING WHEN PARTICIPANTS CHOOSE TO WEAR THE DEVICE. | BULKINESS OF THE MONITOR WAS A NEGATIVE FEATURE OF APPEARANCE OF DIGITAL DEVICE. |
|  | THE KEYBOARD WAS FRUSTRATING/CHALLENGES WITH OXIMETRY TRANSMISSION/DEVICE FAULT/MOBILE SIGNAL LOSS/IMMOBILITY OF THE DEVICE/DIFFICULTIES PLACING THE DEVICE ON THE BODY. | THE REPETITIVE NATURE OF QUESTIONS LEADING TO INDIVIDUALS FILLING OUT MULTIPLE QUESTIONS AT ONE TIME. |
|  | THE SYSTEM WAS PLAGUED BY CONNECTIVITY ERRORS CAUSING ONGOING CONCERN AND FRUSTRATION TO PATIENTS. | PATIENTS EXPERIENCED TECHNICAL ERRORS AND DIFFICULTY IN REVIEWING PREVIOUSLY ENTERED OPEN-TEXT DATA. |
|  | SOME PATIENTS REPORTED FEELING ISOLATED WITH THE MOBILE DEVICE AND FELT THAT THE TOOL COULD BECOME A REPLACEMENT FOR IN PERSON CONSULTATION. | RISK OF ADDICTION TO SMARTPHONES |
|  | RISK OF DANGER OF CELL PHONE RADIATION | RISK OF DISTORTION OF ONGOING RELATIONSHIPS AND COGNITIVE CHANGES |
|  | DATA SECURITY: RISK TO A PERSON USING CERTAIN DEVICES THAT ARE CONNECTED VIA BLUETOOTH, AS WIRELESS COMMUNICATION CAN BE INTERCEPTED BY ELECTROMAGNETIC DEVICES OR HACKED BY CYBER ATTACKERS. | INCREASED PATIENT ANXIETY |
|  | A POTENTIAL BARRIER IS THE COST, SINCE THE USE OF APPS REQUIRES THE PERSON TO USE AN EXPENSIVE SMARTPHONE AND AN INTERNET DATA PLAN | DATA PROTECTION: PROBLEMS WITH SENSITIVE DATA STORAGE BY THE INSTITUTIONS OR GOVERNMENTS WANTING TO STORE HEALTH RECORDS (a. what legal rules might be enforced? b. who owns the data: patients or the device or software owner?) |
