# Supplementary material for: The gaps and challenges in digital health technology use as perceived by patients: a scoping review and narrative meta-synthesis
Source: Front Digit Health. 2025 Mar 27;7:1474956. doi: 10.3389/fdgth.2025.1474956 (PMC11983460; doi:10.3389/fdgth.2025.1474956)
Supplement: Supplementary file 4 [file Table4.docx]

**Supplemental Table 4.** Conceptual Categorization of Reported Findings.

| **ETHICAL ISSUES** | **EDUCATIONAL/TRAINING/LITERACY ISSUES** | **USABILITY ISSUES** | **COMMUNICATION ISSUES** | **FUNCTIONALITY ISSUES** | **TECHNICAL ISSUES** | **ACCESS ISSUES** |
| --- | --- | --- | --- | --- | --- | --- |
| PATIENTS NEED TO FEEL THAT THEY CAN RELY ON THE APP/PRIVACY & SECURITY OF PERSONAL INFORMATION | HEALTH & TECHNOLOGY LITERACY DEFICITS/DIGITAL HEALTH LITERACY & LIFE COACHING | INABILITY TO USE DIGITAL HEALTH TECHNOLOGIES/POOR AWARENESS OF TECHNOLOGY/FEELING INCAPABLE OF USING THE TECHNOLOGY (COMPUTERS OR MOBILE DEVICES) | CHANGE IN PATIENT-PHYSICIAN RELATIONSHIP | ONLY 1 IN 4 REVEALED A QUALITY STANDARD | THE LIMITED BATTERY LIFE OF SMARTPHONES | LACK OF INTERNET OR SMARTPHONE ACCESS |
| PATIENTS NEED TO BE AWARE THAT THE INFORMATION IS COMING FROM A TRUSTWORTHY SOURCE | LACK OF TECHNICAL SKILLS | THE USABILITY OF DHI WAS FEATURED UNDER QUALITY AS SOME FELT THEY WOULD NOT SIGN UP IF IT WAS SLOW OR CUMBERSOME TO REGISTER OR USE IT | LANGUAGE AND CULTURAL BARRIERS IN EFFECTIVE COMMUNICATION AND UNDERSTANDING. | NEED FOR FURTHER DECISION SUPPORT STRUCTURE/SOME WANTED TO SPEAK WITH A CLINICIAN TO CHECK THEIR DECISIONS | LACK OF COMPUTER OR MOBILE EQUIPMENT | INABILITY TO ACCESS AFFORDABLE TECHNOLOGIES DUE TO PROHIBITIVE COSTS INVOLVED |
| THE MAJORITY OF USERS DO NOT UNDERSTAND THE ETHICAL ISSUES ASSOCIATED WITH MOBILE APPS WHICH DO NOT OFFER THE RIGHT TO PRIVACY. | PROBLEMS WITH ENGLISH LITERACY SINCE NOT FIRST LANGUAGE | THE SMARTPHONE WAS CHALLENGING TO USE THAN COMPUTERS | ABSENCE OF NON-VERBAL COMMUNICATION WAS PROBLEMATIC. | PARTICIPANTS FOUND FLAWS IN THE PROMPTS & REMINDER SYSTEM | PATIENTS EXPERIENCED TECHNICAL ERRORS AND DIFFICULTY IN REVIEWING PREVIOUSLY ENTERED OPEN-TEXT DATA. | NEED FOR INDIVIDUAL CULTURALLY TAILORING OF THE PROGRAM, ADDRESSING SUPPORT MECHANISMS AND IMPROVE SITE ACCESSIBILITY/ENHANCING THEIR ENGAGEMENT. |
| THE QUALITY OF HEALTH INFORMATION ACCESSED ONLINE WAS THOUGHT UNRELIABLE & THE POTENTIAL FOR IDENTITY FRAUD MAKE IT DIFFICULT FOR SOME TO TRUST ADVICE FROM VIRTUAL HEALTHCARE PROFESSIONALS. | LACK OF AWARENESS OF THE EXISTENCE OF HEALTH TECHNOLOGY DUE TO NO WIDE PROMOTION | POOR USABILITY OF APPS |  | MANAGE MEDICATIONS |  |  |
| PRIVACY CONCERNS AS A BARRIER TO ACCEPTABILITY SINCE VERY FEW WERE HESITANT ABOUT INFORMATION SECURITY OR ENABLING LOCATION TRACKING | FEARS & FRUSTRATIONS AS A RESULT OF NOT FULLY UNDERSTANDING THE TECHNOLOGIES | LACK OF EXPERIENCE WITH SIMILAR TECHNOLOGIES CREATED WORRIES/FEAR OF DOING SOMETHING WRONG OR GOING OVER DATA ALLOWANCES |  | LOSS OF FINANCIAL INFORMATION |  |  |
| CONCERNS ABOUT SECONDARY USE OF PERSONAL DATA, AS THE USER IS LOCKED WITHIN A LIMITED-OPTIONS ECOSYSTEM OF  DEVICE MANUFACTURERS | LACK OF MOTIVATION TO UNDERSTAND & IMPROVE THEIR HEALTH THROUGH ELECTRONIC DATA | PARTICIPANTS WERE DISSATISFIED WITH THE NEED TO LOG IN EVERY TIME & WAIT TO THE LOADING PROCESS |  | NEED FOR EVIDENCE OF APP EFFECTIVENESS |  |  |
| NO SEAL OR CERTIFICATION THAT MAKES IT EASY FOR THE END-USER TO UNDERSTAND WHICH PRODUCTS USE HIGH INDUSTRY-STANDARD LEVELS OF SECURITY & ARE SAFE TO USE | DIFFICULTIES IN UNDERSTANDING THE RECRUITMENT MESSAGE | THE NEED TO REMEMBER PASSWORDS/FORGOTTEN EMAILS AND PASSWORDS, MULTI-STEP VERIFICATION PROCESSES FOR ACCOUNT |  | LACK OF HUMAN CONTACT MAKES PEOPLE FEEL DISCONNECTED (TELEMEDICINE) |  |  |
| RISK THAT REAL-LIFE DOCTORS WILL ONLY BE AFFORDABLE FOR PATIENTS WITH ADEQUATE INSURANCE OR FINANCIAL RESOURCES WHILE OTHERS WILL BE PREDOMINANTLY TREATED BY AVATARS OR TELEMEDICAL CONSULTANTS | TECHNOLOGY LITERACY BARRIER. | FINDING THE RIGHT APP AT THE RIGHT TIME WHEN PATIENTS DOWNLOADING A MOBILE APP |  | RISK OF DANGER OF CELL PHONE RADIATION |  |  |
| CONCERNS WITH PATIENT-REPORTED MEASURES OF HEALTH DUE TO POTENTIAL INCREASED SUBJECTIVITY | LOW CONFIDENCE IN PATIENT'S ABILITY TO INTERPRET HEALTH DATA ON EHRs OR mhealth APPS/RESULTING IN INCREASED ANXIETY AND CONCERN ABOUT INCORRECT SELF-DIAGNOSIS OR TAKING INAPPROPRIATE STEPS TO SEEKING CARE. | PUSH NOTIFICATIONS WERE RECEIVED TOO SLOWLY OR TOO OFTEN |  | LACK OF CLINICAL ENDORSEMENT |  |  |
| CONFIDENTIALITY & INTEGRITY |  | SOME ASPECTS OF THE APP WERE TOO WORDY OR LENGTHY |  | LACK THE NUANCES FOR HUMAN INTERACTION |  |  |
| DATA REPOSITORY OWNERSHIP |  | INCORPORATING PATIENT PROFILES UP FRONT CAN MAKE THE USER EXPERIENCE MORE PATIENT-CENTERED |  | NEED FOR TAILORED EXPERIENCES |  |  |
| INDIVIDUALS MAY BE HESITANT TO DIVULGE HEALTH RELATED INFO, BECAUSE TEXT-MESSAGES BETWEEN PATIENT & PROVIDERS ARE NOT COVERED BY REGULATIONS SET FORTH BY THE HEALTH INSURANCE PORTABILITY & ACCOUNTABILITY ACT |  | BUILDING IN CONVERSATIONAL ENTITIES, LIKE CHATBOTS, TO ANSWER QUESTIONS IN REAL TIME CAN BE USED TO SIMULATE A MORE INTERACTIVE USER EXPERIENCE |  | TECHNOLOGY WAS VIEWED AS POTENTIALLY DISRUPTIVE OR PURELY FOR ENTERTAINMENT PURPOSES & NOT FOR HEALTHCARE NEEDS |  |  |
| NEED TO NEGOTIATE REGULATORY ISSUES SURROUNDING LICENSING |  | OLDER PATIENTS/AGE CRITERIA |  | THE APP'S GRAPHIC DESIGN LOOKED OUTDATED |  |  |
| USERS CAN BE HESITANT IN SHARING THEIR PERSONAL INFORMATION |  | PERSONALIZATION/USABILITY/FAMILIARITY/COMFORT |  | INAPPLICABILITY OF REMOTE MONITORING FOR ACUTELY ILL PATIENTS (TELEMEDICINE) |  |  |
| CONCERN ABOUT LINKING PARTICIPANTS TO THEIR DIGITAL IDENTITY |  | MANUAL LOGGING & REGISTRATION OF DIFFERENT OBSERVATIONS & MEASUREMENTS |  | DHI WAS A CONSTANT REMINDER OF THEIR FAILURE TO MEET HEALTHY GOALS & WAS THOUGHT TO BE DISCOURAGING |  |  |
| ABUSIVE OR THREATENING BEHAVIOUR DEVELOPED IN VIRTUAL RELATIONSHIPS PREVENTED FROM ENGAGING & ENROLLING |  | THE POTENTIAL FOR PERSONALIZATION/FREQUENT MANUAL INPUT FROM THE USER PERCEIVED AS BURDENSOME & DECREASES INTEREST |  | LACK OF ACCEPTANCE AS A VIABLE OPTION TO MANAGE THEIR CARE WHEN BEING TREATED |  |  |
| SECURE TRANSPORT PROTOCOL |  | LACK OF LONG-TERM ENGAGEMENT/USER ABANDONMENT |  | SEEING NO VALUE IN DHI OFFERED |  |  |
| PATIENT GENERATED DATA |  | TIME COMMITMENT TO HEALTH APPS |  | THE SYSTEM WAS PLAGUED BY CONNECTIVITY ERRORS CAUSING ONGOING CONCERN AND FRUSTRATION TO PATIENTS. |  |  |
| LACK OF CONTROL OVER DATA |  | THE ABILITY TO PROVIDE PERSONALIZED FEEDBACK TAILORED TO THEIR PREFERENCES & CHARACTERISTICS |  | THE REPETITIVE NATURE OF QUESTIONS LEADING TO INDIVIDUALS FILLING OUT MULTIPLE QUESTIONS AT ONE TIME. |  |  |
| USER RESISTANCE TO NEW TECHNOLOGIES |  | LITTLE TIME OR ENTHUSIASM FOR ENGAGING WITH DHIs/PERSONAL LIFE & VALUES |  | ABSENCE OF USER-FRIENDLY FUNCTIONALITIES TO INTERPRET CONSENT DOCUMENTS |  |  |
| LACK OF HUMAN CONTACT MAKES PEOPLE FEEL DISCONNECTED. |  | THERE IS AN INCREASING NUMBER OF APPS. |  |  |  |  |
| NEED FOR REGULATORY FRAMEWORKS |  | SELECTING THE RIGHT DEVICES FROM THE INCREASING NUMBER OF DIGITAL DEVICES ON THE MARKET |  |  |  |  |
| SOME PATIENTS REPORTED FEELING ISOLATED WITH THE MOBILE DEVICE AND FELT THAT THE TOOL COULD BECOME A REPLACEMENT FOR IN PERSON CONSULTATION. |  | PATIENTS NEED TO BE MOTIVATED TO CONSISTENTLY USE THESE TECHNOLOGIES, ADHERE TO MONITORING PROTOCOLS AND ACTIVELY PARTICIPATE IN THEIR OWN CARE. |  |  |  |  |
| A PERCEIVED STIGMA AND EMBARRASSMENT, AFFECTING WHEN PARTICIPANTS CHOOSE TO WEAR THE DEVICE. |  | THE KEYBOARD WAS FRUSTRATING/CHALLENGES WITH OXIMETRY TRANSMISSION/DEVICE FAULT/MOBILE SIGNAL LOSS/IMMOBILITY OF THE DEVICE/DIFFICULTIES PLACING THE DEVICE ON THE BODY. |  |  |  |  |
| CONCERNS WITH INEQUITY IN ACCESS AND USE OF DIGITAL HEALTH SERVICES/BARRIERS RELATING TO ACCESS STABLE INTERNET OR DIGITAL DEVICES/LANGUAGE BARRIERS/DISABILITIES/LOW DIGITAL LITERACY HINDERING ACCESS AND USE. |  | BULKINESS OF THE MONITOR WAS A NEGATIVE FEATURE OF APPEARANCE OF DIGITAL DEVICE. |  |  |  |  |
| CONCERNS WITH COMPROMISED SAFETY/RISK OF MISSED DIAGNOSIS DUE TO THE CLINICIAN'S INABILITY TO PHYSICALLY EXAMINE PATIENTS/MISSING HUMAN CONNECTION WITH THE DOCTOR/A PERCEIVED REDUCTION OF HOLISTIC CARE. |  | POTENTIAL FOR DISCOMFORT OR SKIN IRRITATION FROM PROLONGED WEAR. |  |  |  |  |
| USER COMPLIANCE AND LONG-TERM ADHERENCE TO WEARABLE'S. |  | RELIANCE ON USER INPUT FOR DATA COLLECTION AND ACCURACY/ POTENTIAL FOR MISINTERPRETATION OR INCORRECT INPUT/RELIANCE ON USER ENGAGEMENT AND MOTIVATION TO CONSISTENTLY USE THE APP. |  |  |  |  |
| ETHICAL CONCERNS REGARDING PRIVACY, CONSENT AND POTENTIAL MISUSE OF PATIENT DATA (AI). |  | A POTENTIAL BARRIER IS THE COST, SINCE THE USE OF APPS REQUIRES THE PERSON TO USE AN EXPENSIVE SMARTPHONE AND AN INTERNET DATA PLAN |  |  |  |  |
| LACK OF INTERPERSONAL REASSURANCE |  |  |  |  |  |  |
| RISK OF ADDICTION TO SMARTPHONES |  |  |  |  |  |  |
| RISK OF DISTORTION OF ONGOING RELATIONSHIPS AND COGNITIVE CHANGES |  |  |  |  |  |  |
| DATA PROTECTION: PROBLEMS WITH SENSITIVE DATA STORAGE BY THE INSTITUTIONS OR GOVERNMENTS WANTING TO STORE HEALTH RECORDS (a. what legal rules might be enforced? b. who owns the data: patients or the device or software owner?) |  |  |  |  |  |  |
| DATA SECURITY: RISK TO A PERSON USING CERTAIN DEVICES THAT ARE CONNECTED VIA BLUETOOTH, AS WIRELESS COMMUNICATION CAN BE INTERCEPTED BY ELECTROMAGNETIC DEVICES OR HACKED BY CYBER ATTACKERS. |  |  |  |  |  |  |
| INCREASED PATIENT ANXIETY |  |  |  |  |  |  |
| MOST PAYERS DO NOT COVER THE COST OF HAVING MEDICAL DEVICES OR APPS DUE TO LACK OF CONCLUSIVE DATA |  |  |  |  |  |  |
| INSUFFICIENT SCIENTIFIC EVIDENCES |  |  |  |  |  |  |
