# Supplementary material for: The gaps and challenges in digital health technology use as perceived by patients: a scoping review and narrative meta-synthesis
Source: Front Digit Health. 2025 Mar 27;7:1474956. doi: 10.3389/fdgth.2025.1474956 (PMC11983460; doi:10.3389/fdgth.2025.1474956)
Supplement: Supplementary file 5 [file Table5.docx]

**Supplemental Table 5.** Three Pillars Guiding the Meta-Synthesis and Recommendations.

*4a) Gaps*

| ***A) Digital literacy*** | ***B) Functionality/Usability*** | | ***C)Trust*** |
| --- | --- | --- | --- |
| HEALTH & TECHNOLOGY LITERACY DEFICITS/DIGITAL HEALTH LITERACY & LIFE COACHING. | LACK OF INTERNET OR SMARTPHONE ACCESS | LACK THE NUANCES FOR HUMAN INTERACTION | NEED TO NEGOTIATE REGULATORY ISSUES SURROUNDING LICENSING |
| LACK OF EXPERIENCE WITH SIMILAR TECHNOLOGIES CREATED WORRIES/FEAR OF DOING SOMETHING WRONG OR GOING OVER DATA ALLOWANCES. | LACK OF ACCEPTANCE AS A VIABLE OPTION TO MANAGE THEIR CARE WHEN BEING TREATED | NEED FOR TAILORED EXPERIENCES | PATIENTS NEED TO FEEL THAT THEY CAN RELY ON THE APP/PRIVACY & SECURITY OF PERSONAL INFORMATION |
| LACK OF AWARENESS OF THE EXISTENCE OF HEALTH TECHNOLOGY DUE TO NO WIDE PROMOTION. | LACK OF TECHNICAL SKILLS. | NEED FOR FURTHER DECISION SUPPORT STRUCTURE/SOME WANTED TO SPEAK WITH A CLINICIAN TO CHECK THEIR DECISIONS. | PATIENTS NEED TO BE AWARE THAT THE INFORMATION IS COMING FROM A TRUSTWORTHY SOURCE. |
| LACK OF MOTIVATION TO UNDERSTAND & IMPROVE THEIR HEALTH THROUGH ELECTRONIC DATA | LACK OF COMPUTER OR MOBILE EQUIPMENT | NEED FOR EVIDENCE OF APP EFFECTIVENESS | LACK OF HUMAN CONTACT MAKES PEOPLE FEEL DISCONNECTED (TELEMEDICINE) |
|  | PATIENTS NEED TO BE MOTIVATED TO CONSISTENTLY USE THESE TECHNOLOGIES, ADHERE TO MONITORING PROTOCOLS AND ACTIVELY PARTICIPATE IN THEIR OWN CARE. | LACK OF CLINICAL ENDORSEMENT | LACK OF CONTROL OVER DATA |
|  |  | NEED FOR INDIVIDUAL CULTURAL TAILORING OF THE PROGRAM, ADDRESSING SUPPORT MECHANISMS AND IMPROVING SITE ACCESSIBILITY/ENHANCING THEIR ENGAGEMENT. | AUTHORIZATION & ACCESS CONTROL TECHNOLOGIES |
|  |  |  | NEED FOR REGULATORY FRAMEWORKS. |
|  |  |  | LACK OF INTERPERSONAL REASSURANCE. |
|  |  |  | INSUFFICIENT SCIENTIFIC EVIDENCE |
|  |  |  | MOST PAYERS DO NOT COVER THE COST OF HAVING MEDICAL DEVICES OR APPS DUE TO A LACK OF CONCLUSIVE DATA. |

*4b) Challenges*

| ***A) Digital literacy*** | ***B) Functionality/Usability*** | | | ***C) Trust*** | |
| --- | --- | --- | --- | --- | --- |
| PROBLEMS WITH ENGLISH LITERACY SINCE NOT FIRST LANGUAGE | SELECTING THE RIGHT DEVICES FROM THE INCREASING NUMBER OF DIGITAL DEVICES ON THE MARKET. | THE USABILITY OF DHI WAS FEATURED UNDER QUALITY AS SOME FELT THEY WOULD NOT SIGN UP IF IT WAS SLOW OR CUMBERSOME TO REGISTER OR USE IT | THE SMARTPHONE WAS CHALLENGING TO USE THAN COMPUTERS | USERS CAN BE HESITANT IN SHARING THEIR PERSONAL INFORMATION | CONFIDENTIALITY & INTEGRITY |
| DIFFICULTIES IN UNDERSTANDING THE RECRUITMENT MESSAGE | MANUAL LOGGING & REGISTRATION OF DIFFERENT OBSERVATIONS & MEASUREMENTS | OLDER PATIENTS/AGE CRITERIA | THE LIMITED BATTERY LIFE OF SMARTPHONES | ABUSIVE OR THREATENING BEHAVIOUR DEVELOPED IN VIRTUAL RELATIONSHIPS PREVENTED FROM ENGAGING & ENROLLING | CONCERNS WITH PATIENT-REPORTED MEASURES OF HEALTH DUE TO POTENTIAL INCREASED SUBJECTIVITY |
| FEARS & FRUSTRATIONS AS A RESULT OF NOT FULLY UNDERSTANDING THE TECHNOLOGIES | LACK OF LONG-TERM ENGAGEMENT/USER ABANDONMENT | POOR USABILITY OF APPS | THERE IS AN INCREASING NUMBER OF APPS | THE QUALITY OF HEALTH INFORMATION ACCESSED ONLINE WAS THOUGHT UNRELIABLE & THE POTENTIAL FOR IDENTITY FRAUD MAKE IT DIFFICULT FOR SOME TO TRUST ADVICE FROM VIRTUAL HEALTHCARE PROFESSIONALS. | INDIVIDUALS MAY BE HESITANT TO DIVULGE HEALTH RELATED INFO, BECAUSE TEXT-MESSAGES BETWEEN PATIENT & PROVIDERS ARE NOT COVERED BY REGULATIONS SET FORTH BY THE HEALTH INSURANCE PORTABILITY & ACCOUNTABILITY ACT |
| TECHNOLOGY LITERACY BARRIER. | THE POTENTIAL FOR PERSONALIZATION/FREQUENT MANUAL INPUT FROM THE USER PERCEIVED AS BURDENSOME & DECREASES INTEREST | LOSS OF FINANCIAL INFORMATION | ONLY 1 IN 4 REVEALED A QUALITY STANDARD | THE MAJORITY OF USERS DO NOT UNDERSTAND THE ETHICAL ISSUES ASSOCIATED WITH MOBILE APPS WHICH DO NOT OFFER THE RIGHT TO PRIVACY | CONCERN ABOUT LINKING PARTICIPANTS TO THEIR DIGITAL IDENTITY |
| LANGUAGE AND CULTURAL BARRIERS IN EFFECTIVE COMMUNICATION AND UNDERSTANDING. | INAPPLICABILITY OF REMOTE MONITORING FOR ACUTELY ILL PATIENTS (TELEMEDICINE) | TIME COMMITMENT TO HEALTH APPS | FINDING THE RIGHT APP AT THE RIGHT TIME WHEN PATIENTS DOWNLOADING A MOBILE APP | PRIVACY CONCERNS AS A BARRIER TO ACCEPTABILITY SINCE VERY FEW WERE HESITANT ABOUT INFORMATION SECURITY OR ENABLING LOCATION TRACKING | USER RESISTANCE TO NEW TECHNOLOGIES |
|  | INABILITY TO USE DIGITAL HEALTH TECHNOLOGIES/POOR AWARENESS OF TECHNOLOGY/FEELING INCAPABLE OF USING THE TECHNOLOGY (COMPUTERS OR MOBILE DEVICES) | PARTICIPANTS WERE DISSATISFIED WITH THE NEED TO LOG IN EVERY TIME & WAIT TO THE LOADING PROCESS | PUSH NOTIFICATIONS WERE RECEIVED TOO SLOWLY OR TOO OFTEN | CONCERNS ABOUT SECONDARY USE OF PERSONAL DATA, AS THE USER IS LOCKED WITHIN A LIMITED-OPTIONS ECOSYSTEM OF DEVICE MANUFACTURERS | USER COMPLIANCE AND LONG-TERM ADHERENCE TO WEARABLE'S. |
|  | SEEING NO VALUE IN DHI OFFERED | THE NEED TO REMEMBER PASSWORDS/FORGOTTEN EMAILS AND PASSWORDS, MULTI-STEP VERIFICATION PROCESSES FOR ACCOUNT | THE APP'S GRAPHIC DESIGN LOOKED OUTDATED | NO SEAL OR CERTIFICATION THAT MAKES IT EASY FOR THE END-USER TO UNDERSTAND WHICH PRODUCTS USE HIGH INDUSTRY-STANDARD LEVELS OF SECURITY & ARE SAFE TO USE | SOME PATIENTS REPORTED FEELING ISOLATED WITH THE MOBILE DEVICE AND FELT THAT THE TOOL COULD BECOME A REPLACEMENT FOR IN PERSON CONSULTATION. |
|  | DHI WAS A CONSTANT REMINDER OF THEIR FAILURE TO MEET HEALTHY GOALS & WAS THOUGHT TO BE DISCOURAGING | PARTICIPANTS FOUND FLAWS IN THE PROMPTS & REMINDER SYSTEM | SOME ASPECTS OF THE APP WERE TOO WORDY OR LENGTHY | RISK THAT REAL-LIFE DOCTORS WILL ONLY BE AFFORDABLE FOR PATIENTS WITH ADEQUATE INSURANCE OR FINANCIAL RESOURCES WHILE OTHERS WILL BE PREDOMINANTLY TREATED BY AVATARS OR TELEMEDICAL CONSULTANTS | CONCERNS WITH INEQUITY IN ACCESS AND USE OF DIGITAL HEALTH SERVICES/BARRIERS RELATING TO ACCESS STABLE INTERNET OR DIGITAL DEVICES/LANGUAGE BARRIERS/DISABILITIES/LOW DIGITAL LITERACY HINDERING ACCESS AND USE. |
|  | TECHNOLOGY WAS VIEWED AS POTENTIALLY DISRUPTIVE OR PURELY FOR ENTERTAINMENT PURPOSES & NOT FOR HEALTHCARE NEEDS | BUILDING IN CONVERSATIONAL ENTITIES, LIKE CHATBOTS, TO ANSWER QUESTIONS IN REAL TIME CAN BE USED TO SIMULATE A MORE INTERACTIVE USER EXPERIENCE | THE ABILITY TO PROVIDE PERSONALIZED FEEDBACK TAILORED TO THEIR PREFERENCES & CHARACTERISTICS | PATIENT GENERATED DATA | A PERCEIVED STIGMA AND EMBARRASSMENT, AFFECTING WHEN PARTICIPANTS CHOOSE TO WEAR THE DEVICE. |
|  | INABILITY TO ACCESS AFFORDABLE TECHNOLOGIES DUE TO PROHIBITIVE COSTS INVOLVED | LITTLE TIME OR ENTHUSIASM FOR ENGAGING WITH DHIs/PERSONAL LIFE & VALUES | INCORPORATING PATIENT PROFILES UP FRONT CAN MAKE THE USER EXPERIENCE MORE PATIENT-CENTERED | SECURE TRANSPORT PROTOCOL | CONCERNS WITH COMPROMISED SAFETY/RISK OF MISSED DIAGNOSIS DUE TO THE CLINICIAN'S INABILITY TO PHYSICALLY EXAMINE PATIENTS/ABSENCE OF NON-VERBAL COMMUNICATION WAS PROBLEMATIC. CONCERN ABOUT THE IMPACT OF DIGITAL HEALTH SERVICES ON PATIENT-PHYSICIAN RELATIONSHIP/MISSING HUMAN CONNECTION WITH THE DOCTOR/A PERCEIVED REDUCTION OF HOLISTIC CARE. |
|  | PERSONALIZATION/USABILITY/FAMILIARITY/COMFORT | MANAGE MEDICATIONS | CHANGE IN  PATIENT-PHYSICIAN RELATIONSHIP | DATA REPOSITORY OWNERSHIP | RELIANCE ON USER INPUT FOR DATA COLLECTION AND ACCURACY/ POTENTIAL FOR MISINTERPRETATION OR INCORRECT INPUT/RELIANCE ON USER ENGAGEMENT AND MOTIVATION TO CONSISTENTLY USE THE APP. |
|  | THE KEYBOARD WAS FRUSTRATING/CHALLENGES WITH OXIMETRY TRANSMISSION/DEVICE FAULT/MOBILE SIGNAL LOSS/IMMOBILITY OF THE DEVICE/DIFFICULTIES PLACING THE DEVICE ON THE BODY. | THE REPETITIVE NATURE OF QUESTIONS LEADING TO INDIVIDUALS FILLING OUT MULTIPLE QUESTIONS AT ONE TIME. | BULKINESS OF THE MONITOR WAS A NEGATIVE FEATURE OF APPEARANCE OF DIGITAL DEVICE. | RISK OF ADDICTION TO SMARTPHONES | LOW CONFIDENCE IN PATIENT'S ABILITY TO INTERPRET HEALTH DATA ON EHRs OR mhealth APPS/RESULTING IN INCREASED ANXIETY AND CONCERN ABOUT INCORRECT SELF-DIAGNOSIS OR TAKING INAPPROPRIATE STEPS TO SEEKING CARE. |
|  | THE SYSTEM WAS PLAGUED BY CONNECTIVITY ERRORS CAUSING ONGOING CONCERN AND FRUSTRATION TO PATIENTS. | PATIENTS EXPERIENCED TECHNICAL ERRORS AND DIFFICULTY IN REVIEWING PREVIOUSLY ENTERED OPEN-TEXT DATA. | POTENTIAL FOR DISCOMFORT OR SKIN IRRITATION FROM PROLONGED WEAR. | INCREASED PATIENT ANXIETY | RISK OF DISTORTION OF ONGOING RELATIONSHIPS AND COGNITIVE CHANGES |
|  | RISK OF DANGER OF CELL PHONE RADIATION | A POTENTIAL BARRIER IS THE COST, SINCE THE USE OF APPS REQUIRES THE PERSON TO USE AN EXPENSIVE SMARTPHONE AND AN INTERNET DATA PLAN |  | DATA PROTECTION: PROBLEMS WITH SENSITIVE DATA STORAGE BY THE INSTITUTIONS OR GOVERNMENTS WANTING TO STORE HEALTH RECORDS (a. what legal rules might be enforced? b. who owns the data: patients or the device or software owner?) | DATA SECURITY: RISK TO A PERSON USING CERTAIN DEVICES THAT ARE CONNECTED VIA BLUETOOTH, AS WIRELESS COMMUNICATION CAN BE INTERCEPTED BY ELECTROMAGNETIC DEVICES OR HACKED BY CYBER ATTACKERS. |
